# Bis-Pyrene Photo-Switch Open- and Closed-Form Differently Bind to ds-DNA, ds-RNA and Serum Albumin and Reveal Light-Induced Bioactivity

**DOI:** 10.3390/ijms22094916

**Published:** 2021-05-06

**Authors:** Iva Orehovec, Marija Matković, Isabela Pehar, Dragomira Majhen, Ivo Piantanida

**Affiliations:** 1 Division of Organic Chemistry and Biochemistry, Ruđer Bošković Institute, Bijenička Cesta 54, 10000 Zagreb, Croatia; Iva.Orehovec@irb.hr (I.O.); Marija.Matkovic@irb.hr (M.M.); 2 Division of Molecular Biology, Ruđer Bošković Institute, Bijenička Cesta 54, 10000 Zagreb, Croatia; Isabela.Pehar@irb.hr (I.P.); Dragomira.Majhen@irb.hr (D.M.)

**Keywords:** photo-switch, diarylethene, pyrene, DNA, RNA binding, BSA binding, cell uptake, cytotoxicity

## Abstract

Newly designed and synthesized diarylethene (DAE) derivatives with aliphatic amine sidearms and one with two pyrenes, revealed excellent photo-switching property of central DAE core in MeOH and water. The only exception was bis-pyrene analogue, its DAE core very readily photochemically closed, but reversible opening completely hampered by aromatic stacking interaction of pyrene(s) with cyclic DAE. In this process, pyrene fluorescence showed to be a reliable monitoring method, an open form characterized by strong emission at 480 nm (typical for pyrene-aggregate), while closed form emitted weakly at 400 nm (typical for pyrene-DAE quenching). Only open DAE-bis-pyrene form interacted measurably with ds-DNA/RNA by flexible insertion in polynucleotide grooves, while self-stacked closed form did not bind to DNA/RNA. For the same steric reasons, flexible open DAE-bis-pyrene form was bound to at least three different binding sites at bovine serum albumin (BSA), while rigid, self-stacked closed form interacted dominantly with only one BSA site. Preliminary screening of antiproliferative activity against human lung carcinoma cell line A549 revealed that all DAE-derivatives are non-toxic. However, bis-pyrene analogue efficiently entered cells and located in the cytoplasm, whereby irradiation by light (315–400 nm) resulted in a strong, photo-induced cytotoxic effect, typical for pyrene-related singlet oxygen species production.

## 1. Introduction

Synthetic small molecules targeting DNA, RNA or proteins by non-covalent interactions are of utmost scientific and biomedical interest [1], among which dyes attract attention not only because spectrophotometric probing is essential in the biomedical research [2,3], but also due to theranostic applications by combining, e.g., fluorimetric probing with biological (therapeutic) activity [4,5].

The biological action of small molecules controlled by outer stimuli has become of high interest rather recently; for instance, pH-controlled DNA-targeting systems [6,7,8], or photo-induced formation of bioactive and fluorescent molecules [9,10].

Particularly, reversible photo-control of biological systems by molecular photo-switches attracted considerable attention [11,12]. In respect to DNA-controlled non-covalent binders, the pioneering work of Feringa et al. showed how small molecule non-covalently bound to ds-DNA can upon photo-induced reversible chemical transformation influence DNA structure [13]. Similarly, it was shown that DNA helicity can be somewhat changed by photo-switchable small molecules [14]. Lately, significant progress was made by Ihmels et al., demonstrating a wide range of heterocyclic systems, which can be finely tuned [15,16,17,18]. In addition, very recently new photo-switchable non-covalently interacting optoepigenetic tools for conditional modulation of nucleosomal DNA accessibility were reported [19]. Likewise, photo-dependent melting of unmodified DNA using a photosensitive intercalator as a new and generic tool for photo-reversible assembly of DNA nanostructures has been described [20]. Further, photo-controlled capture and release of DNA and proteins were demonstrated by host-guest constructs [21]. Low molecular weight photo-switch incorporated into DNA structure was able to control two single-stranded DNAs to reversibly intertwine into ds-DNA [22].

Particularly, diarylethenes (DAE) drew our attention due to the high conversion rate (mostly close to 100%), at variance to lower conversion rates of other photo-switches (e.g., diaza mostly being about 70–80%, although some analogues showed promising increase [23]). In addition, some diarylethene derivatives showed highly fluorescent “closed” forms [24], very promising property for the applications like super-resolution microscopy [25].

Inspired by considerable advancement in photo-switch recognition of bio-related systems, we aimed for the development of a new, fluorescent, low molecular weight conjugate able to non-covalently interact with DNA, RNA and/or protein, whereby recognition properties could be controlled by a photo-switchable process. The photo-induced bioactivity of the novel small molecule would be screened on cell lines.

**Scheme 1:**
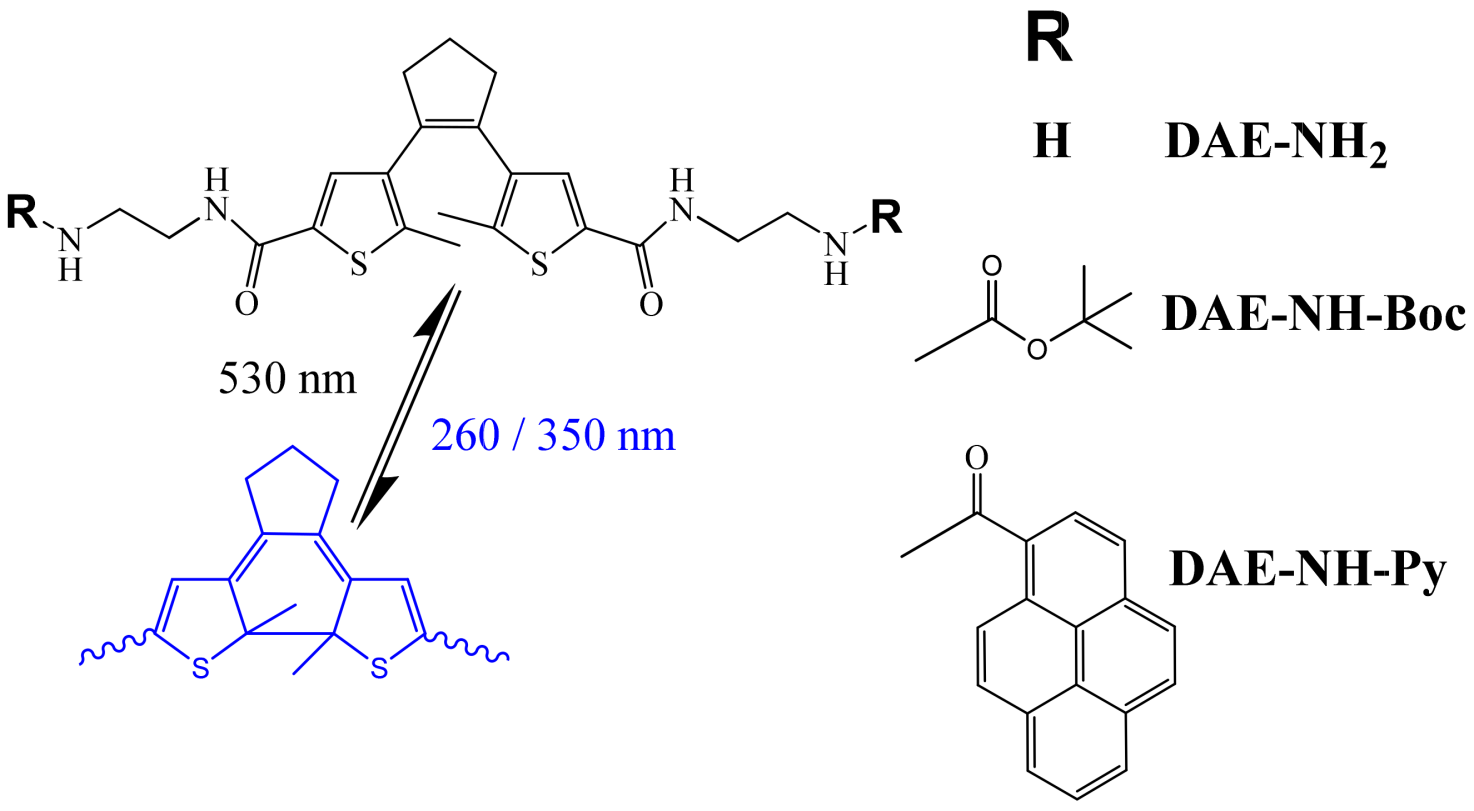
Studied diarylethene (DAE) compounds. Note reversible photo-switching; irradiation by UV light results in cyclization (BLUE), which can be reversed by irradiation in the visible range (530 nm).

Starting from DAE-derivative targeting ds-DNAs studied by Feringa [13], and with the aim to increase the structural impact of the cyclisation-decyclisation process of DAE, we decided to shorten the linker between DAE and aliphatic amine by omitting benzene rings used by Feringa [13] (Scheme 1, DAE-NH_2_). Further, we introduced pyrenes at the termini of both arms (Scheme 1, DAE-NH-Py), to allow sensitive monitoring of the photo-switching process by fluorescence. Namely, pyrene fluorescence is very sensitive to the microenvironment [2] and also can form excimers characterized by very specific emission [2,26]. Therefore, pyrenes were intensively used for various probing of DNA/RNA/proteins [2,26,27], as well as theragnostic agents [5,28]. Moreover, some pyrene derivatives were applicable in photodynamic therapy (PDT) under two-photon absorption (PTA) conditions [29]. In this work pyrenes in DAE-NH-Py are expected to sense fine differences within DNA/RNA/protein binding site and also contribute to the binding by hydrophobic and aromatic stacking interactions. Furthermore, pyrene(s) can also stack by aromatic interactions on the “closed”, planar form of DAE, but not to “open”, non-planar form of DAE, in this way directly controlling the small molecule geometry and flexibility.

As targeted biomacromolecules, we have chosen ds-DNA/RNA of various secondary structure (Table S1, Supplementary Materials), thus differing in properties of binding sites (minor or major grooves and eventually intercalation): namely, we previously showed interesting applications of pyrene fluorophore by relying on its interactions with the DNA or RNA grooves [30] or combined with switch on and off of pyrene excimer [6,7,8]. In addition, since some pyrene analogues tended to prefer proteins over ds-DNA [31], we studied interactions of new compounds with the representative protein, i.e., bovine serum albumin (BSA), the most abundant protein in blood plasma responsible for the transport of many small molecules [32,33], which is characterized by up to eight different binding sites [34].

For comparison reasons we also studied analogue with *Boc*-protected amino groups (Scheme 1, DAE-NH-Boc), whereby *Boc*-group may be considered similarly hydrophobic as pyrene, but void of aromaticity and being globular instead of planar.

In this work, we showed by a set of spectrophotometric methods (UV/vis, fluorescence, circular dichroism and DNA/RNA thermal denaturation experiments) that photo-induced switching (Scheme 1) very efficiently works for new compounds under well-defined conditions. In addition, closed and open form of bis-pyrene DAE-NH-Py very differently interacted with both, DNA/RNA and protein (BSA). Preliminary screening on human tumour cell line showed efficient cellular uptake of DAE-NH-Py, accompanied by negligible toxicity in dark, but showed increased biological activity under light excitation of pyrenes.

## 2. Results

### 2.1. Synthesis

**Scheme 2:**
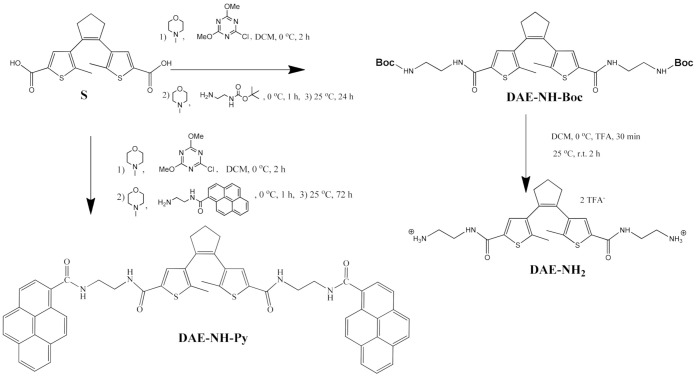
Synthesis of referent compounds DAE-NH-Boc and DAE-NH_2_ and targeted pyrene derivative DAE-NH-Py.

The core diarylethene switch S was prepared in four steps according to the procedure already described in the literature [35] with some modifications in the synthetic process (Supplementary Materials, Scheme S1). Further, we extended core-switch DAE with flexible side-arms N-Boc-ethylenediamine (DAE-NH-Boc, Scheme 2), by application of mild coupling method well known in peptide chemistry [36]. Deprotection by common procedure [37] resulted in diamine derivative DAE-NH_2_ (Scheme 2).

Attempt to prepare desired pyrene derivative DAE-NH-Py by simple coupling of DAE-NH_2_ with 1-aminopyrene was unsuccessful due to complete degradation of the product during purification. Therefore, we opted for a parallel route, preparing DAE-NH-Py by direct reaction of S with separately prepared N-(2-aminoethyl)pyrene-1-carboxamide (Supplementary Materials; Scheme S4), whereby the final DAE-NH-Py was obtained in a rather low yield due to losses during purification (Scheme 2).

### 2.2. Spectrophotometric Characterisation

Compound DAE-NH-Boc was dissolved in MeOH at 10^−3^ mol dm^−3^, DAE-NH_2_ was dissolved in mQ H_2_O at 10^−3^ mol dm^−3^ and DAE-NH-Py was dissolved in DMSO at 10^−3^ mol dm^−3^. These stock solutions were stored at +4 °C and daily working aliquots kept at +25 °C. The experiments were performed in buffer solution (sodium cacodylate buffer, *I* = 50 mM, pH = 7.0) and in MeOH.

DAE-NH-Boc, DAE-NH_2_ and DAE-NH-Py absorbance in aqueous buffered solutions or MeOH solutions of compounds were proportional to their concentration up to 10 µM (Supplementary Materials, Figures S1–S8), corresponding UV/Vis data given in Table 1.

**Figure 1:**
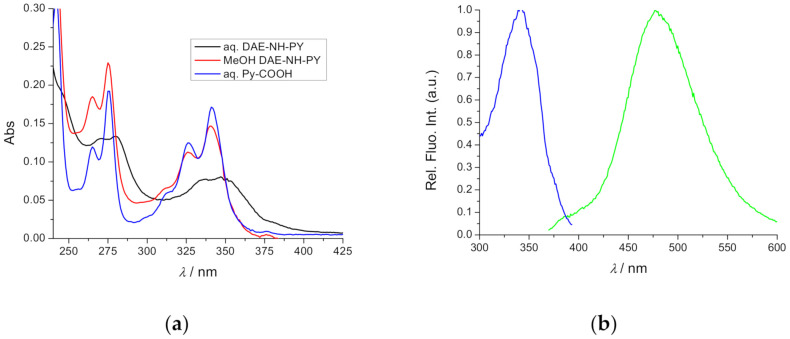
(**a**) UV/Vis spectra of DAE-NH-Py(*c* = 5.0 × 10^−6^ M) in MeOH (▬) and in aqueous buffer (▬) at pH 7.0, sodium cacodylate buffer, *I* = 50 mM (1% DMSO), compared to referent 1-pyrene-carboxylic acid (▬, starting material) in at pH 7.0, sodium cacodylate buffer, *I* = 50 mM. (**b**) Emission (▬, λ_exc_ = 348 nm) and excitation (▬, λ_em_ = 478 nm) spectrum of DAE-NH-Py (*c* = 1.0 × 10^−6^ M) at pH 7.0, sodium cacodylate buffer, *I* = 50 mM (1% DMSO).

Comparison of UV/Vis spectra of pyrene analogue DAE-NH-Py collected in aqueous solution and MeOH showed distinct differences (Figure 1a). The DAE-NH-Py spectrum in MeOH showed sharp and well-defined maxima, very similar to referent pyrene-carboxylic acid (Figure 1a, Py-COOH) and typical of free pyrene moiety [2]. Dissimilarly, DAE-NH-Py aqueous solution UV/Vis spectrum showed strong hypochromic (32%) and bathochromic (+Δλ = 12 nm) effect in respect to MeOH-solution and referent Py-COOH, which can be attributed to the intramolecular stacking interactions of aromatic subunits [2,26].

The pyrene analogue also exhibited fluorescence emission with a maximum at 478 nm (Figure 1b), whereas the corresponding excitation spectrum (Figure 1b) agreed well with UV/Vis spectrum (Figure 1a), confirming the involvement of the same chromophore (pyrene). Since the emission of pyrene in water is characterized by three sharp emission maxima around 400 nm [2], here observed strong bathochromic shift of emission is characteristic for pyrene excimer (500 nm) [26] or eventually aromatically stacked pyrene with some other aromatic moiety, e.g., DAE.

### 2.3. Photochemistry

Diarylethene (DAE) reversible photochemistry (Scheme 1) relies on photo-cyclisation of DAE upon irradiation by UV light, whereby formation of cyclic product is monitored by the appearance of new absorption maximum at about 530 nm. The irradiation of cyclic DAE by visible light (around 530 nm) causes ring-opening, again monitored by decrease and eventually the disappearance of 530 absorption maximum.

For better quantification of applied light, we used a photoreactor (Luzchem, LZC-ICH2), equipped with four side-mounted LZC-UVB lamps (in total 32 W, 254–315 nm) for cyclisation reaction and four side-mounted LZC-420 lamps (in total 32 W, range 400–700 nm) for de-cyclisation reaction. Solutions were irradiated in standard 1 cm pathlength quartz cuvettes, which allowed instant measurement of UV/Vis or fluorescence spectra.

#### 2.3.1. Photo-Controlled Switching of DAE-NH-Boc, DAE-NH_2_ and DAE-NH-Py

**Figure 2:**
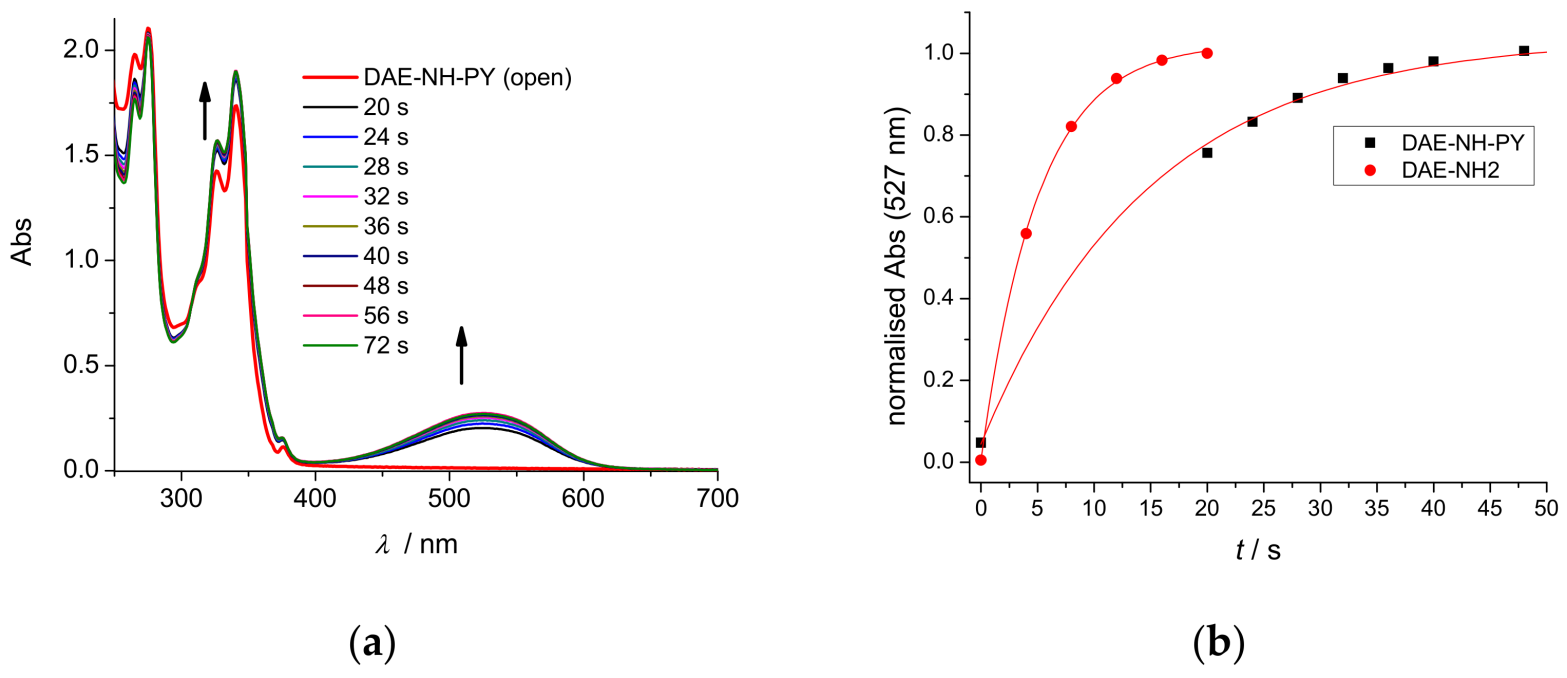
(**a**) Changes in UV/Vis spectra of DAE-NH-Py (*c* = 1.0 × 10^−4^ M) during 32 W lamp, 254–315 nm range irradiation (*t*_tot_. = 72.00 s) in MeOH (4.3% DMSO); (**b**) Normalized Abs changes for DAE-NH-Py and DAE-NH_2_ at λ = 527 nm as a function of irradiation time.

All compounds showed excellent solubility in MeOH, which allowed the concentration-dependent study of the photo-cyclisation process upon irradiation with UV light and monitoring the appearance of λ_max_ = 527 nm, specific for cyclic product. At concentrations c < 0.1 mM cyclisation of all compounds was completed within 12–50 s (Figure 2, Supplementary Materials Figures S9–S17); however, at *c* = 1 mM cyclisation was significantly slower (up to two orders of magnitude) and at *c* = 10 mM cyclisation was completely hampered.

Such concentration-dependent effect can be attributed to two different causes: (a) due to large *ε* values in UV spectra, at high *c*(compound) >1 mM the only fraction of photons reaches reactive species, reaction eventually at 10 mM even reaching zero-order kinetics [38] (b) aggregation of aromatic compounds, which by intermolecular non-covalent interactions inhibited photo-induced cyclisation. Latter was additionally supported by a detailed comparison of cyclisation kinetics of DAE-NH-Py and DAE-NH_2_ (Figure 2b), whereby former bis-pyrene analogue photo-conversion was slower, suggesting that important impact of aggregation-prone pyrene units on the photo-cyclisation process.

The photo-cyclisation process upon irradiation with UV light also worked in aqueous solutions, whereby systematic variation of MeOH/water ratio did not influence the kinetics of reaction (Supplementary Materials, Figure S18).

Comparison of open and closed from of DAE-NH-Py UV/Vis spectra (Figure 2a) revealed several differences; one was the appearance of the band at λ = 527 nm specific for the closed form, but also systematic absorbance increase within 300–350 nm range is observed, attributed to the change of pyrene chromophore caused by cyclisation of DAE core.

**Figure 3:**
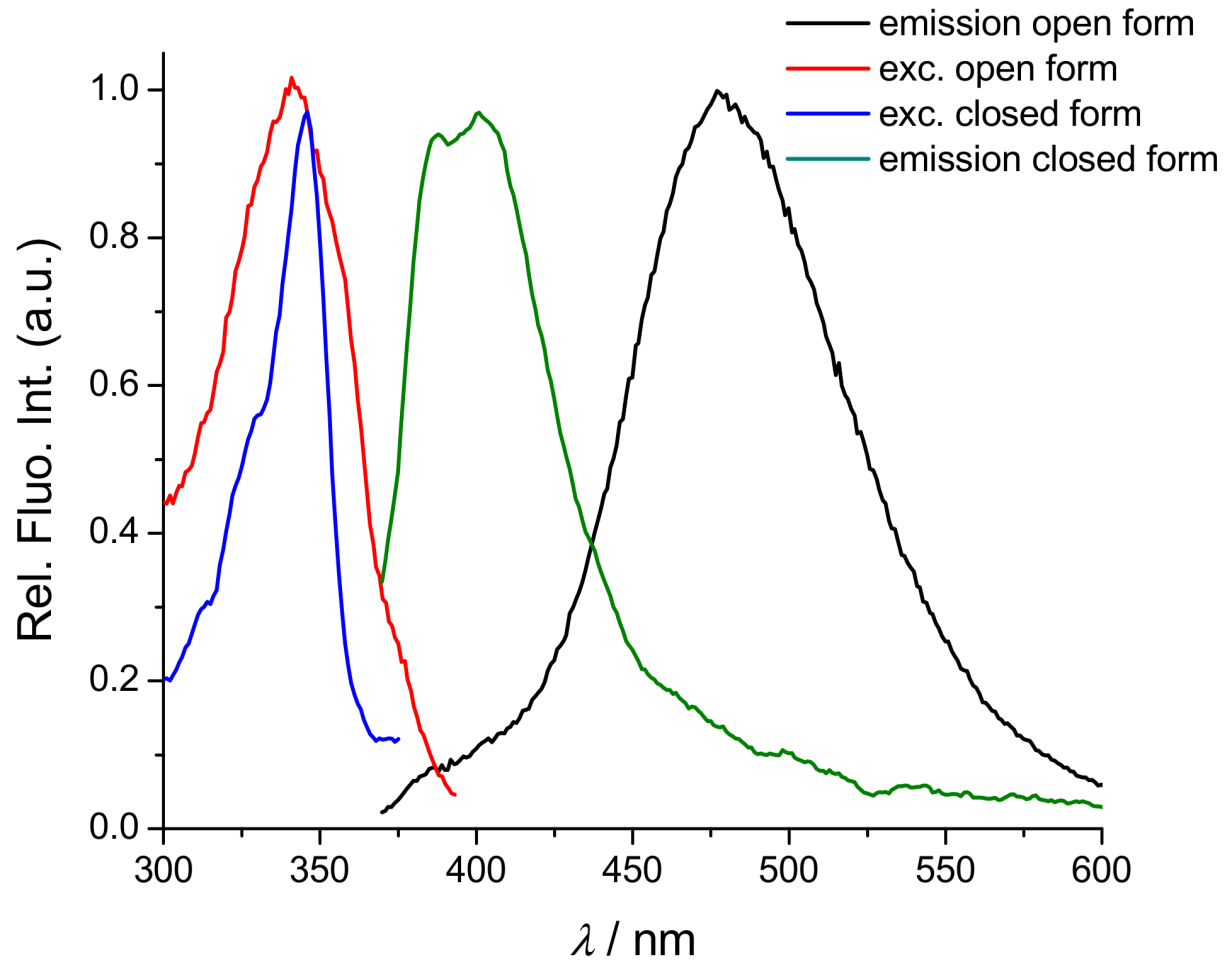
Normalized fluorescence spectra of DAE-NH-Py (*c* = 1.0 × 10^−6^ M) at pH 7.0, sodium cacodylate buffer, *I* = 0.05 M (1% DMSO): open DAE-NH-Py: Emission (▬, λ_exc_ = 348 nm) and excitation (▬, λ_em_ = 478 nm) and closed DAE-NH-Py: Emission (spectrum multiplied by 20; ▬, λ_exc_ = 348 nm) and excitation (▬, λ_em_ = 400 nm) spectrum.

Particularly interesting was the change of DAE-NH-Py fluorescence upon photo-cyclisation (Figure 3). Starting spectrum of open DAE-NH-Py (characterized by emission at 480 nm, typical for pyrene excimer or aggregate [26] within seconds of UV irradiation strongly hypsochromically shifted (−Δλ = 80 nm) and decreased in intensity 20-fold. The emission spectrum of closed DAE-NH-Py resembled very closely to the emission of a single pyrene chromophore, strongly quenched by aromatic interaction with some other aryl moiety [2,26]—likely intramolecularly stacked with closed DAE form. The UV irradiation and subsequent DAE closure did not affect significantly the excitation spectrum of pyrene, proving that pyrene did not undergo any photochemical change.

**Figure 4:**
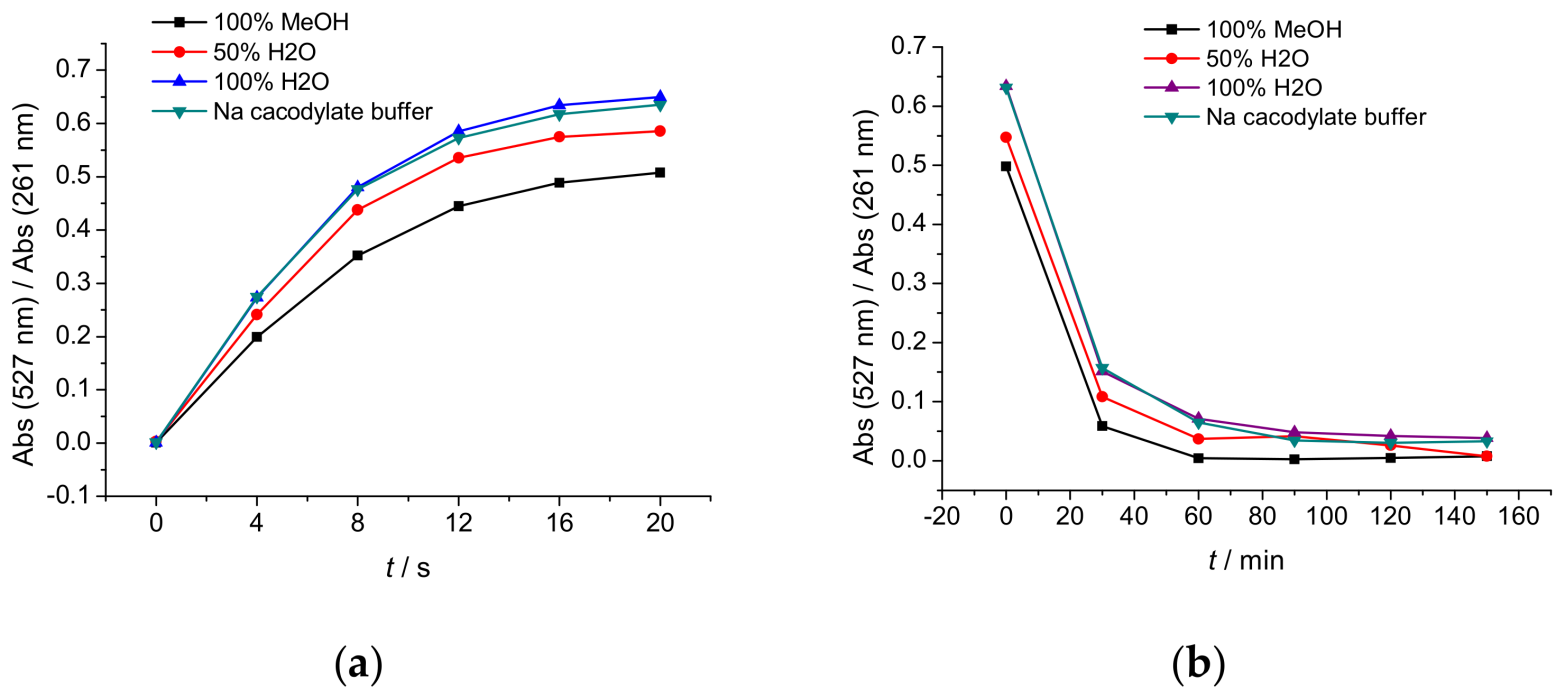
Measurements of DAE-NH_2_ (*c* = 1.0 × 10^−4^ M) at MeOH, 50% H_2_O in MeOH, H_2_O and Na cacodylate buffer (pH = 7.0, I = 0.05 M) showing A_527_/A_261_ ratios during (**a**) irradiation with 64 W, range 254–315 nm lamps, as a function of irradiation time (*t*_tot_. = 20.00 s), (**b**) irradiation with 64 W, range 400–700 nm lamps, as a function of irradiation time (*t*_tot_. = 150.00 min).

Once prepared, the cyclic (closed) form of DAE-NH_2_ can be submitted to the reverse process by irradiation with visible light, such de-cyclisation process again monitored at λ_max_ = 527 nm (Figure 4b). Due to the lower energy of light implemented at longer wavelengths, complete de-cyclisation took about 60–150 min, again independent on a MeOH/water ratio.

**Figure 5:**
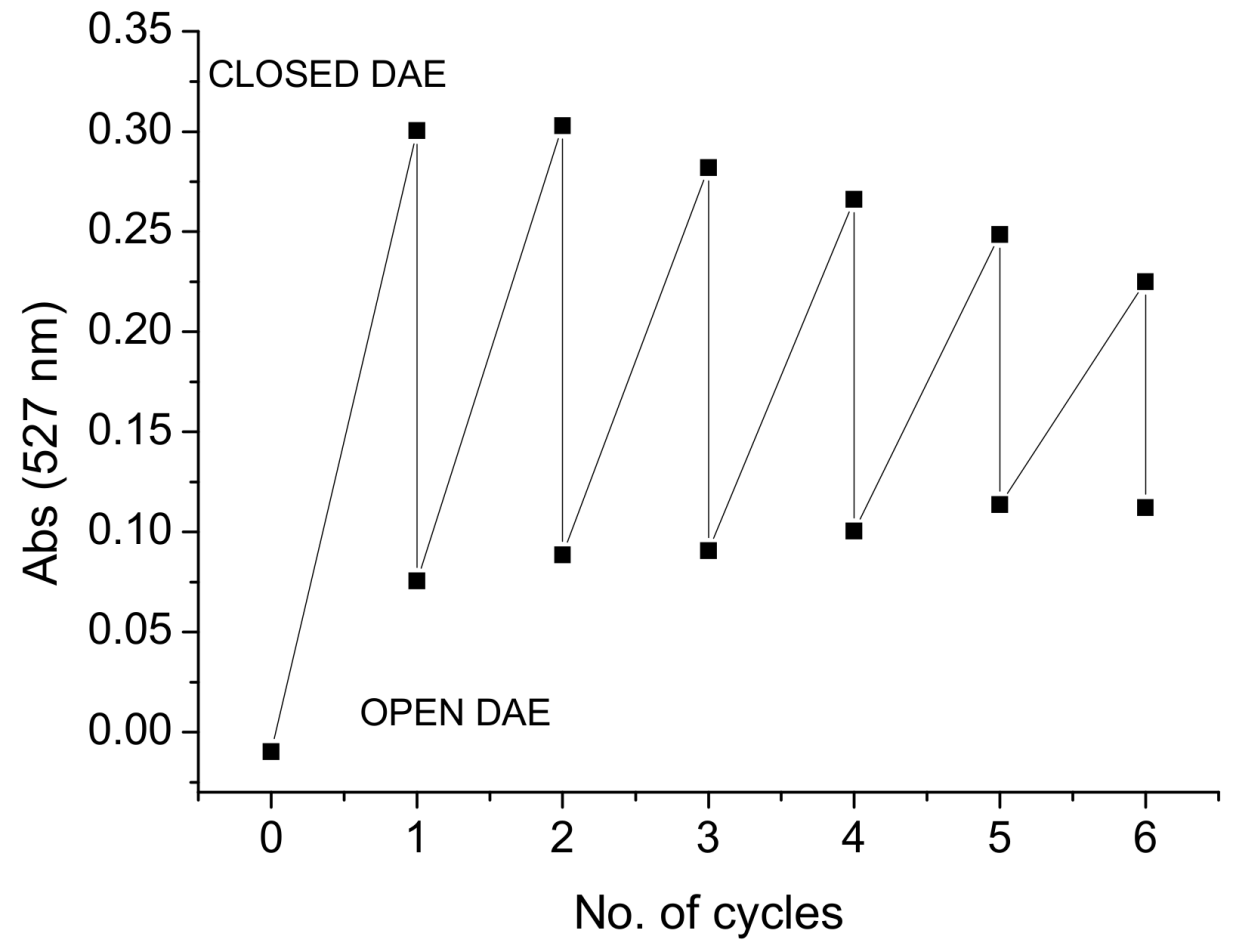
Changes at λ = 527 nm (specific for cyclic DAE form) during 6 repetitive “open-close” irradiations of DAE-NH-Py (*c* = 1.0 × 10^−4^ M), for cyclisation 32 W, range 254–315 nm lamps; for de-cyclisation 32 W, range 400–700 nm lamps, done in MeOH/4.3% DMSO. Note the decrease in conversion rate in both closed DAE and open DAE form, proportional to the number of cycles.

Further, we tested the conversion efficiency of the cyclization–decyclization process in repetitive cycles (Figure 5). The conversion rate decreased with a number of cycles similarly as noted for close analogue of DAE-NH_2_ in corresponding spectrophotometric experiments [13].

Study in the aqueous environment (water and buffer pH 7 system) revealed that, at variance to efficient de-cyclisation of referent DAE-NH_2_ (Figure 4) and also efficient de-cyclisation of hydrophobic DAE-NH-Boc (Supplementary Materials, Figure S13 right), a de-cyclisation process of bis-pyrene DAE-NH-Py was almost completely hampered (Supplementary Materials, Figure S17). This can be correlated to slower kinetics of pyrene-analogue in MeOH (Figure 2, right), which in water progressed to complete blocking of DAE-ring opening, likely due to intensive inter- or intra-molecular interactions of pyrene subunits with DAE-core in cyclic form.

### 2.4. Interactions with DNA/RNA

In all experiments, stock solutions of acyclic and cyclic forms of DAE-NH_2_ and DAE-NH-Py were prepared in mM concentration and then further diluted in buffer pH 7 before the experiment.

#### 2.4.1. Thermal Denaturation of ds-DNA/RNA

Non-covalent binding of small molecules to ds-polynucleotides usually increases the thermal stability of the ds-helices, thus resulting in an increased T_m_ value and this increase (∆T_m_) can (in corroboration with other methods, like circular dichroism or viscometry or NMR) be related to the various binding modes [39]. For example, if pyrene compounds intercalate into the ds-DNA, ∆T_m_ > 2–5 °C can be expected, whereas the binding of pyrenes within the polynucleotide groove should have a negligible stabilizing effect for ds-helix [30].

**Figure 6:**
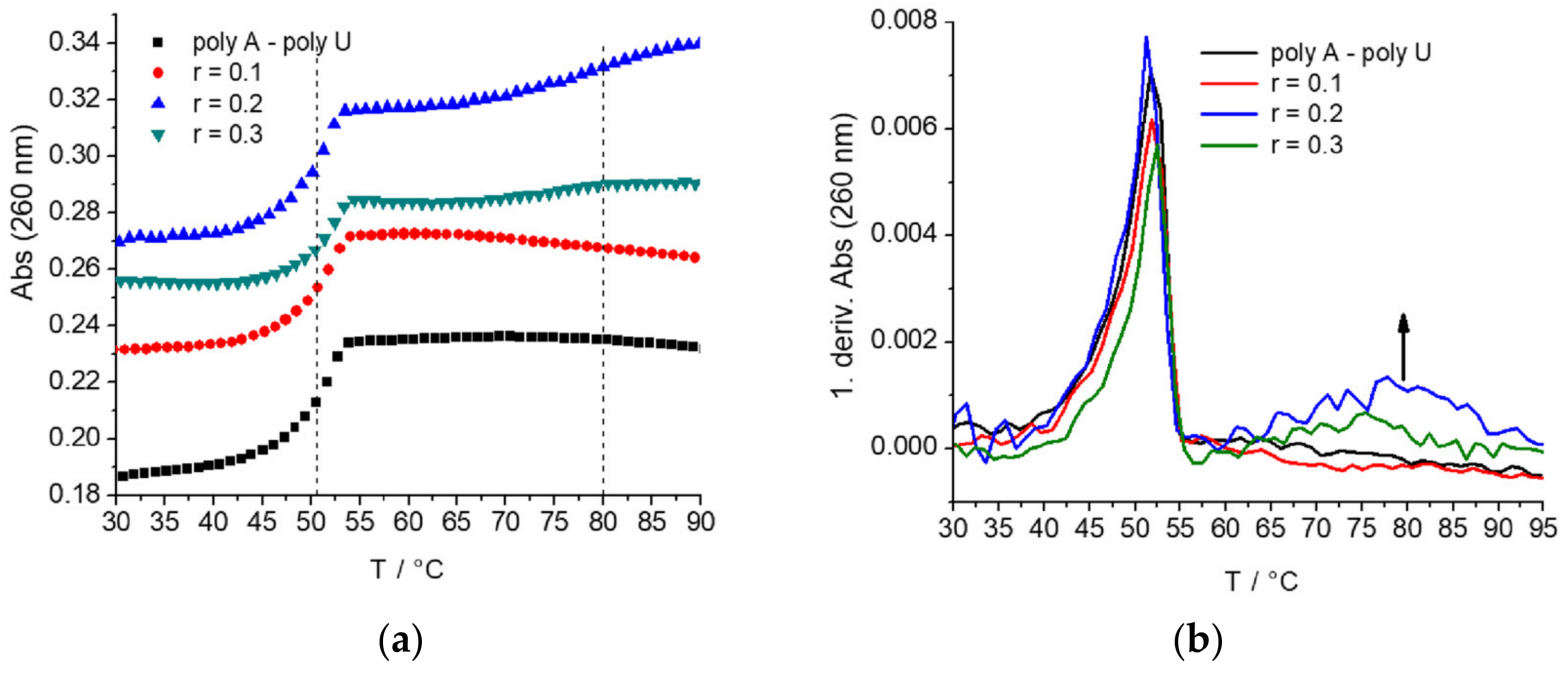
(**a**) Thermal denaturation of poly A—poly U (*c* = 2.0 × 10^−5^ M), upon addition r = 0.1, r = 0.2, r = 0.3, ([compound/[polynucleotide]) of DAE-NH-Py (open form) at pH 7.0 (buffer sodium cacodylate, *I* = 0.05 M)/0.5% DMSO, (**b**) The 1. derivative of absorbance on temperature.

The addition of referent open form of DAE-NH_2_ to any ds-DNA/RNA did not have any impact on the thermal stability of studied polynucleotides, as well as the addition of DAE-NH-Py to ct-DNA. However, the addition of DAE-NH-Py at ratio r = 0.2–0.3 to AU-RNA resulted in the appearance of a second transition at a significantly higher temperature (Figure 6, Table 2). Intriguingly, the first transition at about 52 °C, characteristic for a free AU-RNA, remained and attempt to completely saturate AU-RNA by adding more DAE-NH-Py caused precipitation-like changes. Thus, it seems that at the given ratio r = 0.2–0.3 part of available ds-RNA binding sites was strongly stabilized by DAE-NH-Py, while a simultaneously large portion of RNA remains free of a small molecule. Taking into account the given ratios (r = 0.2–0.3), it seems that DAE-NH-Py aggregates within RNA major groove into dimers. That would also agree with the lack of stabilization of ct-DNA since both DNA grooves lack depth and hydrophobicity of RNA major groove (Supplementary Materials Table S1).

The addition of a “closed” DAE-NH-Py form to both, ds-DNA or ds-RNA did not yield any thermal stabilization of polynucleotides (Table 2). Obviously, photo-cyclisation of DAE-NH-Py prevented the formation of assumed aggregate inside ds-RNA major groove, likely due to intensive stacking of pyrene(s) to cyclic DAE (as discussed at the end of Section 2.3.1).

#### 2.4.2. Circular Dichroism (CD) Experiments

Close analogue of DAE-NH_2_ [13] in the open form was in equilibrium between two dynamic helices of opposite sign, thus having no distinctive CD spectrum. Photochemical ring-closure locks the conformation of the switch in the fixed chirality, however, still not showing a measurable intrinsic CD spectrum due to the equimolar enantiomeric ratio [13].

**Figure 7:**
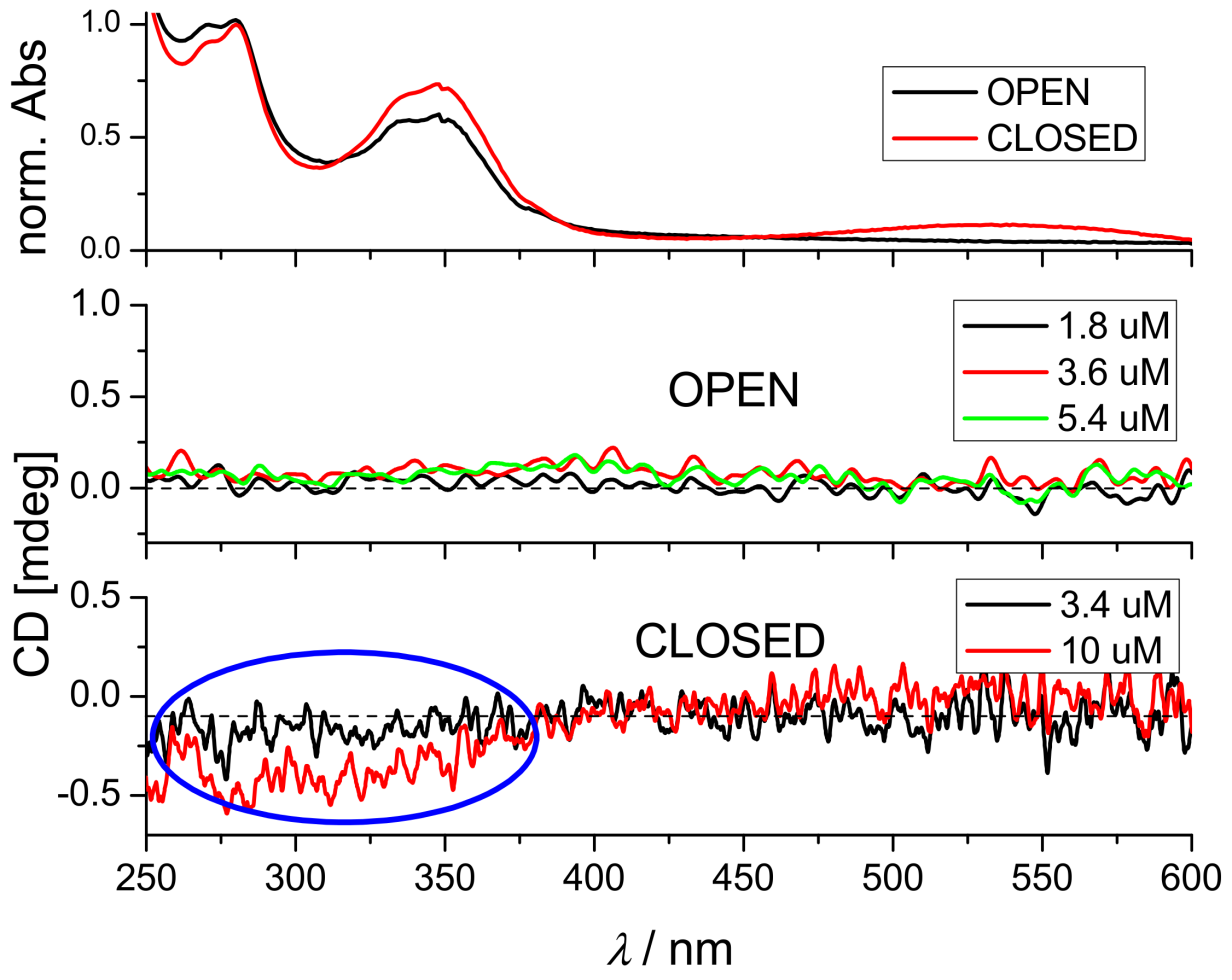
CD spectra of DAE-NH-Py (*c* = 1.8–10 × 10^−6^ M) in an open and closed form, overlaid with corresponding UV/Vis spectra (normalized). Done at pH 7.0, buffer sodium cacodylate, *I* = 0.05 M.

We collected CD spectra of DAE-NH-Py in both, open and closed form (Figure 7), intriguingly closed form showing weak negative CD band at 300–350 nm range, characteristic for pyrene absorption. Since pyrenes, as well as their aliphatic linkers, are not chiral, observed weak CD band suggests that at least one pyrene is in the interaction with DAE core, most likely by stacking interactions (see comments to Figure 2 and Figure 3), which would result in the induced chirality.

**Figure 8:**
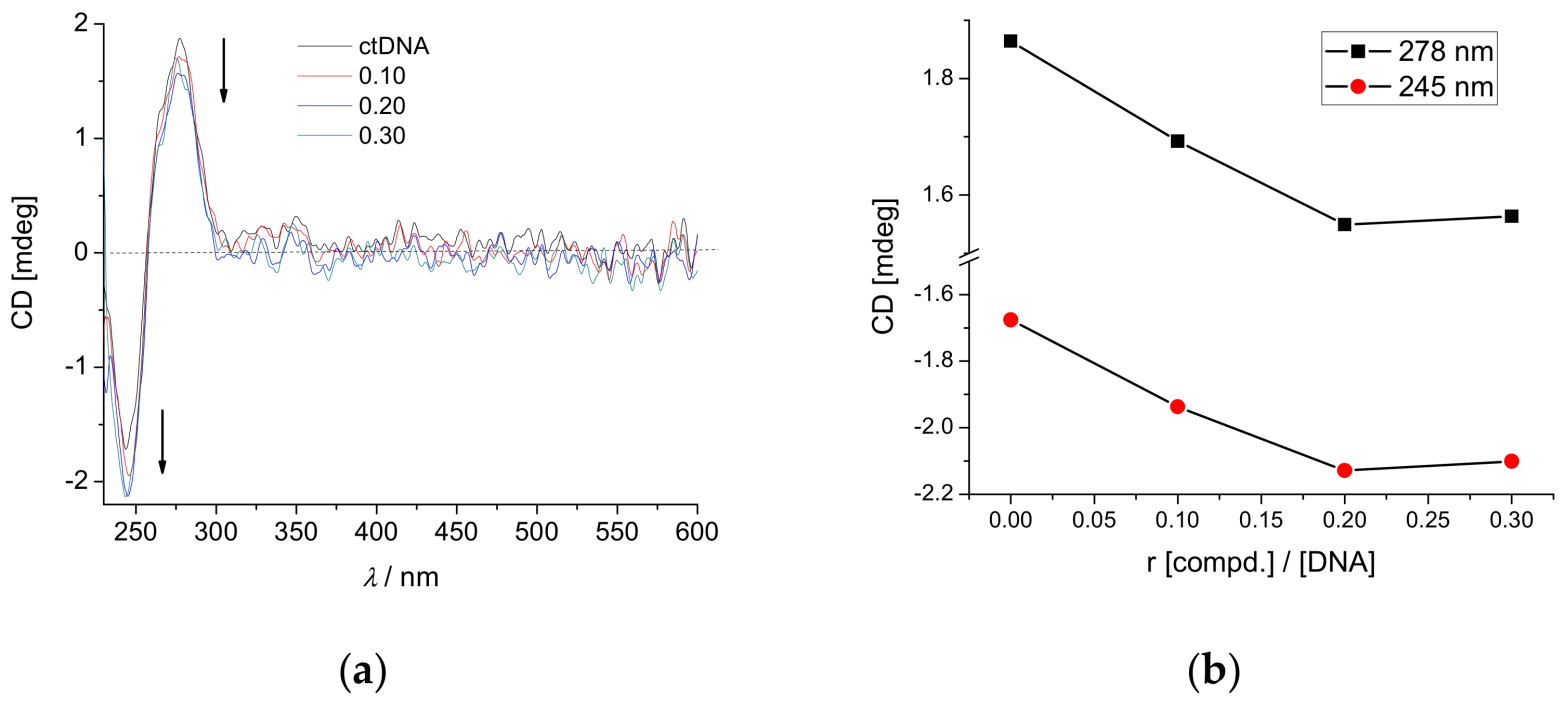
(**a**) CD titration of ct-DNA (*c* = 2 × 10^−5^ M) with DAE-NH-Py closed at molar ratios r = [compound]/[DNA]; (**b**) dependence of CD maxima at 245 nm and 278 nm on a ratio r. Done at pH 7.0, buffer sodium cacodylate, *I* = 0.05 M.

The addition of DAE-NH-Py to ds-DNA caused only a minor decrease of CD bands at 245 nm and 275 nm (Figure 8a, attributed to the minor unwinding of ds-DNA), which reached saturation at ratio r = 0.2 (Figure 8b). No induced CD (ICD) bands >300 nm were observed, thus pointing out that pyrenes are not uniformly oriented in respect to DNA chiral axis [40].

**Figure 9:**
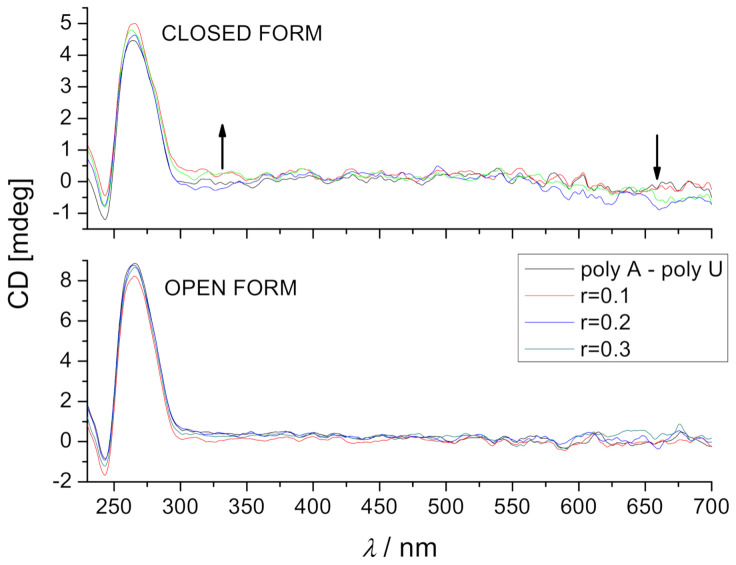
CD titration of poly A—poly U (*c* = 2 × 10^−5^ M) with open and closed form of DAE-NH-Py at molar ratios r = [compound]/[RNA]. Done at pH 7.0, buffer sodium cacodylate, *I* = 0.05 M.

Differently, the addition of closed DAE-NH-Py to AU-RNA induced a very weak ICD band change at 300–350 nm (Figure 9, up), likely due to the ICD band of pyrene. Intriguingly, open DAE-NH-Py did not cause such change in the CD spectrum of AU-RNA (Figure 9, down).

Analogous experiments with open DAE-NH_2_ did not change CD spectra of studied DNA/RNA (Supplementary Materials, Figure S22), whereas closed DAE-NH_2_ somewhat changed the CD spectrum of only poly A—poly U but no changes were observed for ct-DNA. These results are at variance to previously reported for close analogue DAE-phenyl-NH_2_ [13], where binding to ds-DNAs resulted in weak but measurable ICD bands at 350 nm and 570 nm, characterized as DAE-phenyl-core uniformly oriented in the respect of ds-DNA helical axis. Most likely extension of DAE with phenyl [13] yielded a longer aromatic system, which then uniformly positioned chromophore within DNA binding site, thus yielding ICD band(s).

#### 2.4.3. Fluorimetric Titrations

Pyrene analogue DAE-NH-Py is characterized by fluorescence emission sensitive to the microenvironment, as demonstrated by open DAE-NH-Py emitting strong emission at 480 nm, while closed DAE-NH-Py emits 20-fold weaker emission at about 400 nm (Figure 3). Based on that we expected that open and closed DAE-NH-Py will interact quite differently with ds-DNA or ds-RNA and their emission will change accordingly.

**Figure 10:**
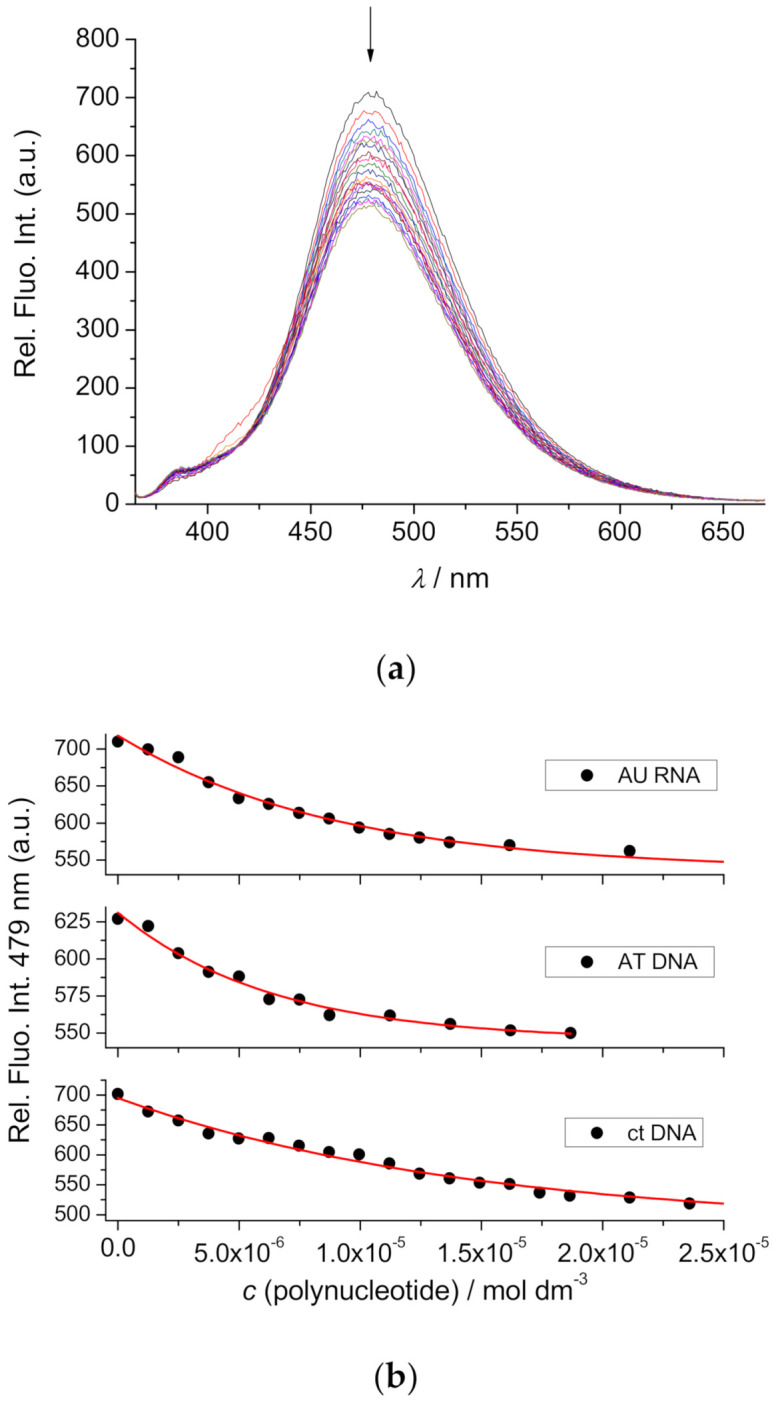
(**a**) Changes in fluorescence spectrum of DAE-NH-Py open (*c* = 1.0 × 10^−6^ M) upon titration with ct-DNA; (**b**) Dependence of emission intensity at λ_max_ = 479 nm on c(polynucleotide), data fitted to Scatchard Equation [42] (▬). Done at pH = 7.0, buffer sodium cacodylate, *I* = 0.05 M, 0.04 % DMSO.

Addition of the most ds-DNA/RNA to open DAE-NH-Py resulted in quenching of fluorescence for about 10–25% (Figure 10), the only exception being GC-DNA (Supplementary Materials, Figure S26), which did not influence open DAE-NH-Py emission. Structural specificity of GC-DNA in respect to other used ds-DNA/RNAs is sterically blocked minor groove by protruding amino groups of guanine [41], which obviously hampered deep insertion of pyrenes into the hydrophobic groove, leaving them exposed to the aqueous environment and therefore not changing their fluorescence.

The addition of any ds-DNA/RNA did not change the fluorescence of closed DAE-NH-Py (Supplementary Materials, Figure S27). The closed form seems to be sterically locked by intramolecular stacking interactions of pyrene(s) and cyclic DAE, thus, forming a highly rigid, aryl-stacked system, which does not fit into any DNA or RNA groove.

Processing of fluorimetric titration data (Figure 10) by means of the Scatchard equation [42] allowed the determination of binding constants (Table 3). The binding affinities are similar, the almost order of magnitude stronger binding to AT-DNA in respect to ct-DNA probably due to the 48% of GC-basepairs in ct-DNA, in which minor groove binding is hampered by protruding amino groups of guanine [41].

### 2.5. Interactions with BSA

**Figure 11:**
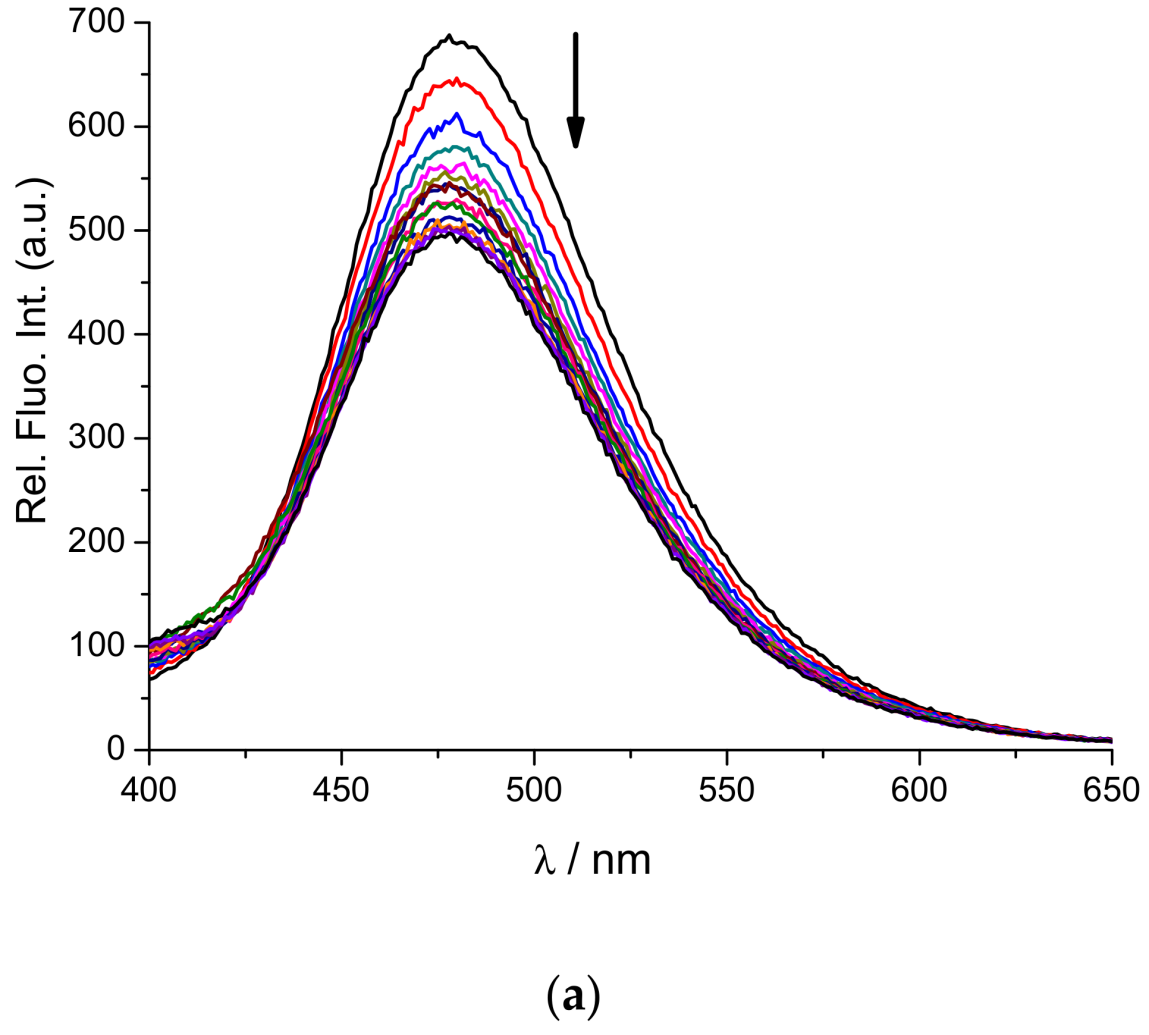

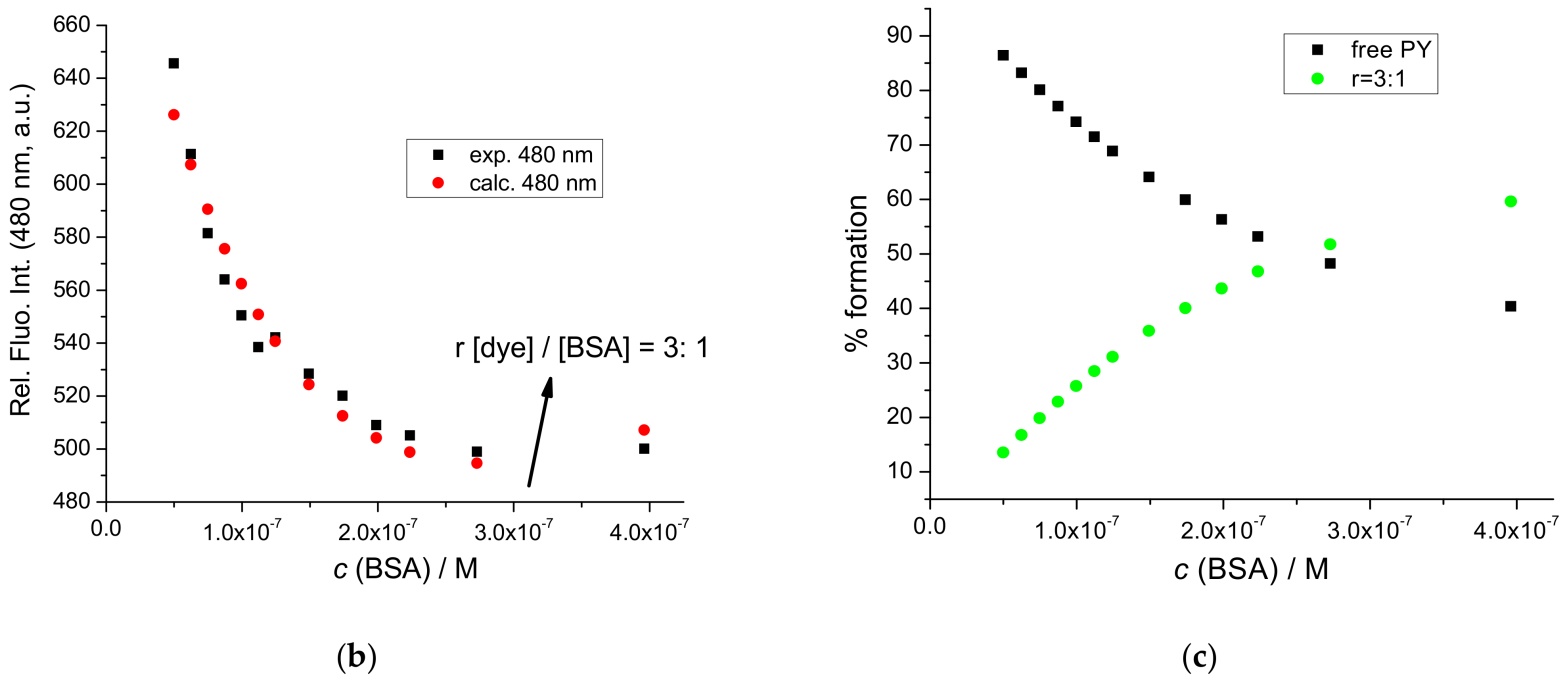
(**a**) Fluorimetric titration of DAE-NH-Py open (*c* = 1 × 10^−6^ M; λ_exc_ = 342 nm) with BSA; (**b**) Dependence of emission at λ_max_ = 480 nm on *c*(BSA): experimental (■ ) and HypSpec2014 [43] calculated data (●); (**c**) Distribution of spectroscopically active species dependent on a *c*(BSA) based on HypSpec2014 processing [43], free DAE-NH-Py open (■ ); 3:1 complex (●). Done at pH 7.0, buffer sodium cacodylate, *I* = 0.05 M.

**Figure 12:**
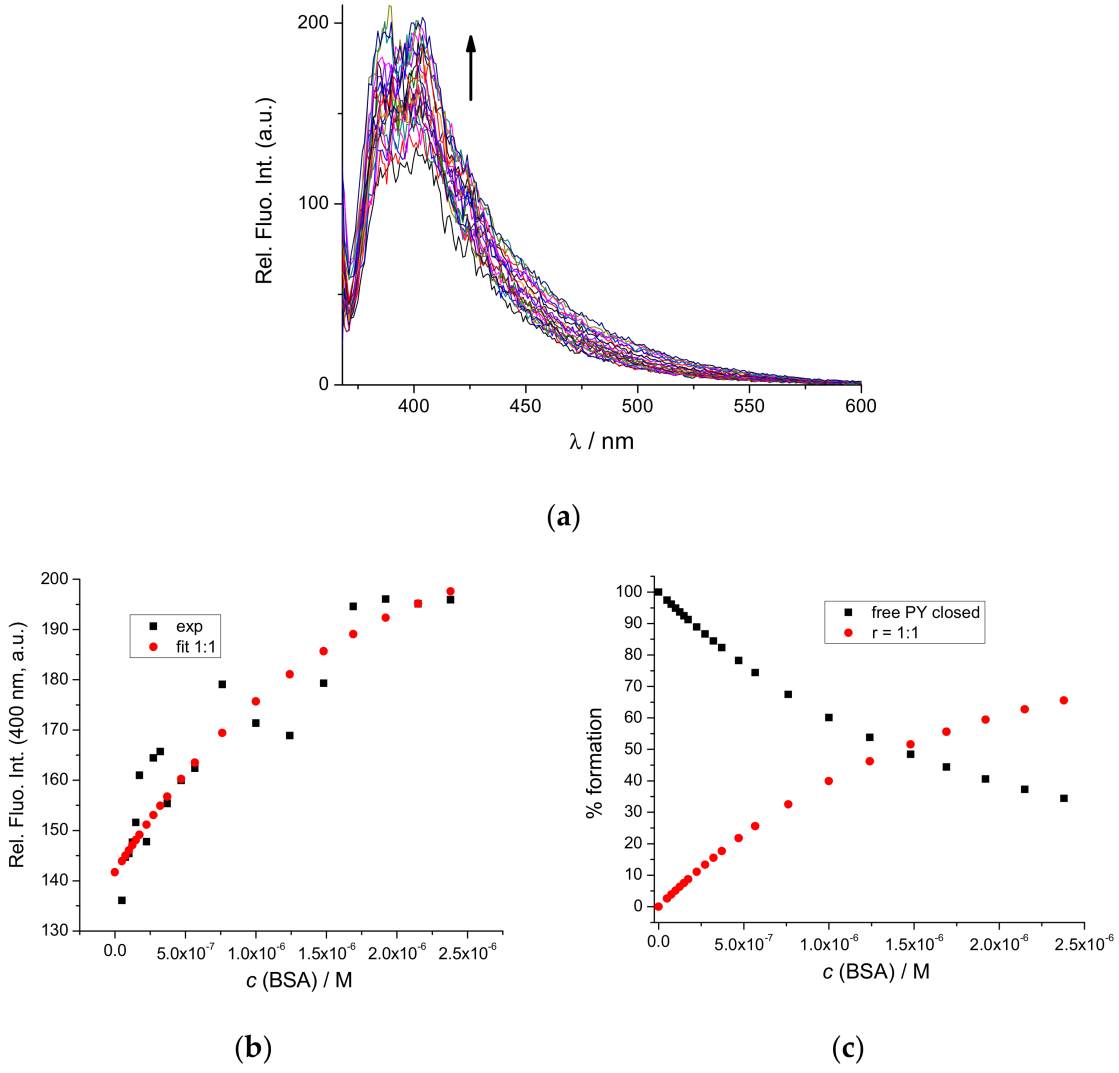
(**a**) Fluorimetric titration of DAE-NH-Py closed (*c* = 1 × 10^−6^ M; λ_exc_ = 342 nm) with BSA; (**b**) Dependence of emission at λ_max_ = 400 nm on *c*(BSA): experimental (■ ) and HypSpec2014 [43] calculated data (●); (**c**) Distribution of spectroscopically active species dependent on a *c*(BSA) based on HypSpec2014 processing [43], free DAE-NH-Py closed (■ ); 1:1 complex (●). Done at pH 7.0, buffer sodium cacodylate, *I* = 0.05 M.

The addition of BSA to DAE-NH-Py resulted in different changes of pyrene emission, strongly dependent on a DAE state (open vs. closed). Namely, emission of pyrene excimer (λ_max_ = 480 nm) DAE-NH-Py open was quenched, reaching the end of a titration at ratio r[dye]/[BSA] ~ 3:1 (Figure 11); at variance to DAE-NH-Py closed, which showed emission increase (λ_max_ = 400 nm, Figure 12) only at large excess of dye over BSA.

Since BSA has multiple binding sites for small molecules and thus, DAE-NH-Py can bind to protein in several different stoichiometries, titration data were processed by nonlinear regression analysis using the HypSpec2014 program [43], designed for multivariate analysis of complex systems.

Analysis of DAE-NH-Py open titration data gave the best fit for the formation of r[DAE-NH-Py open]/[BSA] = 3:1 complex (Figure 11b) with calculated cumulative binding constant logβ_3:1_ = 19 ± 0.7 M^−3^, which agreed well with the distribution of spectroscopically active species (Figure 11c), approaching >70% of complex formed. Statistically, at large excess of BSA over dye (r_[dye]/[BSA]_ < 0.01), 2:1 and 1:1 complex stoichiometries should also be present; however, such high concentrations of BSA are not biologically relevant. We also tried to fit titration data to coexisting 2:1 and 3:1 dye/BSA complexes and got logβ_2:1_ = 11 ± 1.6 M^−2^; logβ_3:1_ = 18 ± 0.9 M^−3^ with a very low percentage of 2:1 complex (thus, large error of logβ_2:1_), whereas model containing 1:1, 2:1 and 3:1 stoichiometry was not possible to fit to experimental data. Thus, at given titration conditions, a 3:1 stoichiometry complex is the dominant species.

The structural meaning of [DAE-NH-Py open]/[BSA] ~ 3:1 complex implies that dye is bound to the three different binding sites on BSA with similar affinity (β_3:1_ = K_s1_ × K_s2_ × K_s3_). A detailed determination which binding sites are targeted would require an elaborate set of competition experiments with particular small molecules known to specifically target some BSA binding site (see Figure 1 in ref. [34]), which is out of the scope of this work.

Oppositely to DAE-NH-Py open, analysis of DAE-NH-Py closed titration data gave the best fit (Figure 12b) for the formation of r[DAE-NH-Py closed]/[BSA] = 1:1 complex with binding constant logK_s1:1_ = 6.0 ± 0.5 M^−1^, which agreed well with the distribution of spectroscopically active species (Figure 11c), approaching >70% of complex formed. Attempts to fit experimental data to any higher stoichiometry (2:1 or 3:1) failed, thus, clearly stressing that only one type of complex is formed.

Such difference in binding stoichiometries and affinities between the open and closed form of DAE-NH-Py can be attributed to the larger flexibility of DAE in open form, which, therefore, easily accommodates within several different binding sites on BSA. The closed form seems to be sterically locked by intramolecular stacking interactions of pyrene(s) and cyclic DAE, thus forming a highly rigid, aryl-stacked system, which binds efficiently in only one BSA binding site, hence giving 1:1 stoichiometry. According to the literature, most BSA/HSA ligands are of low molecular weight (Mw < 500), except bilirubin and hemin (Mw > 700), which bind to the IB:FA1 position of HSA. This binding site is characterized by a deep hydrophobic cleft [34,44], which would by size agree with a stacked DAE-NH-Py closed system.

### 2.6. Biological Experiments

It has been reported that many pyrene derivatives exhibit intriguing biorelevant interactions [2], and even in some cases show intracellular organelle recognition (e.g., mitochondrial) [29]. Additionally, under UV irradiation some pyrenes produce singlet oxygen-related species, thus, having photodynamic therapy (PDT) effect.

**Figure 13:**
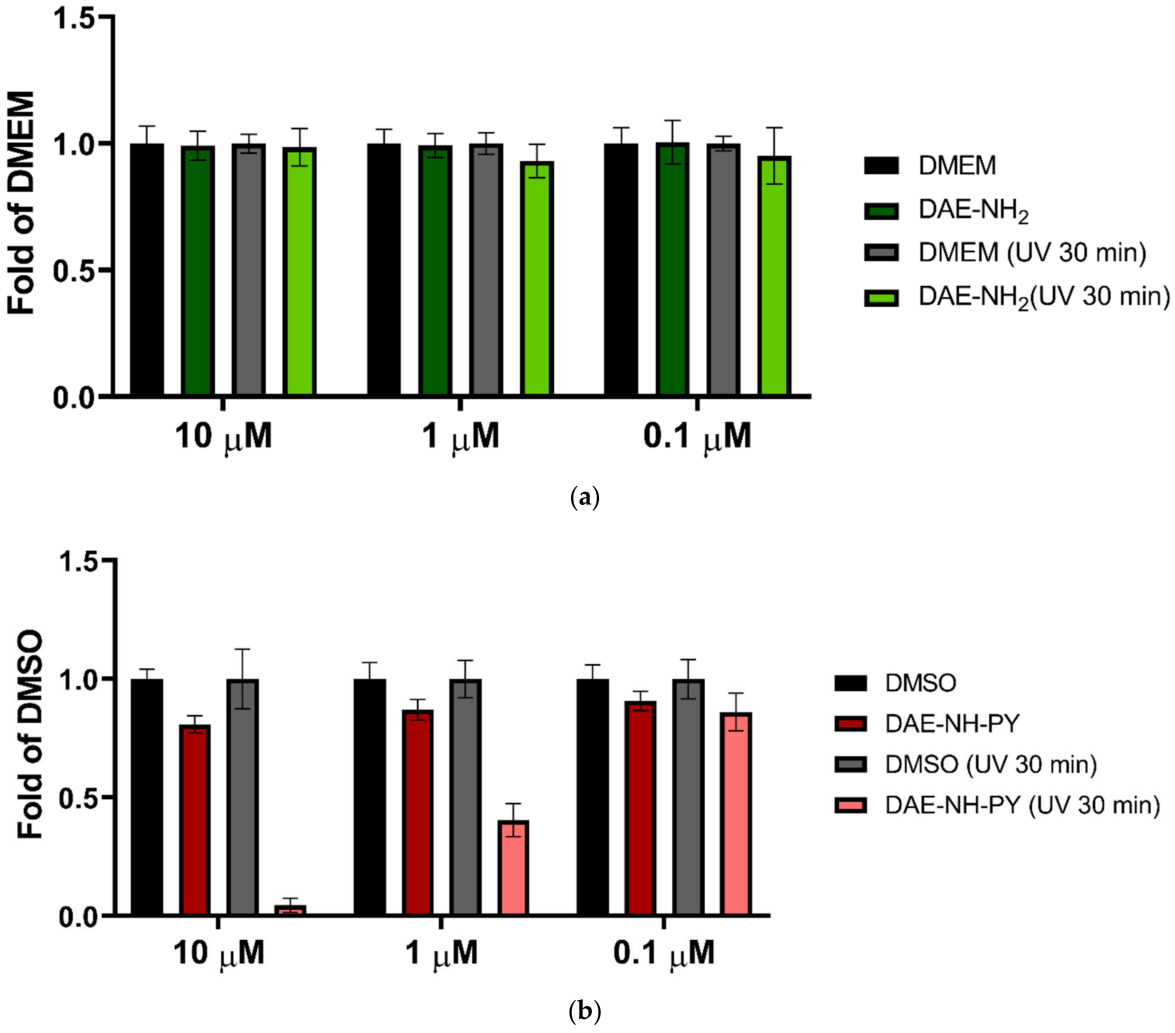
Cell survival of A549 cells treated with DAE-NH_2_ (**a**) or DAE-NH-Py (**b**), with or without UV-A light irradiation (30 min each of three days of cell incubation). Control samples are cells treated with DMSO for DAE-NH-Py, or DMEM for DAE-NH_2_. Representative data of three independent experiments which yielded similar results are shown. Data are presented as relative to the control samples ± SD, made in three replicates.

For that reason, we preliminarily screened cytotoxic activity of DAE-NH-Py and referent DAE-NH_2_ by the MTT assay (for 72 h) against human lung carcinoma (A549) cell line (Figure 13). Tested cells were in two parallel experiments either kept in dark or irradiated by UV-A light in the photoreactor (Luzchem, LZC-ICH2), equipped with eight overhead-mounted LZC-UVA lamps (in total 64 W, 315–400 nm), latter irradiation performed for 30 min on the 1st, 2nd and 3rd day. Results showed that both compounds are non-toxic even at the highest concentration if not exposed to light, referent DAE-NH_2_ being also non-toxic even at prolonged light irradiation (Figure 13a). However, DAE-NH-Py upon 30 min irradiation exhibited concentration-dependent cytotoxic activity (Figure 13b, UV 30 min), measurable as low as at 1 µM.

**Figure 14:**
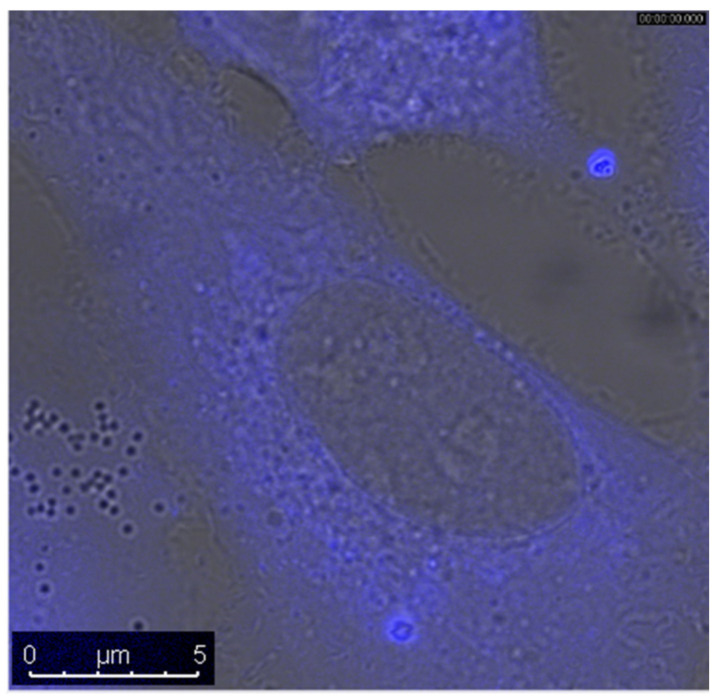
Uptake and localization of DAE-NH-Py (*c* = 10 µM) in live A549 cells. Cells were treated with DAE-NH-Py for 90 min at 37 °C, the surplus of the dye washed with DMEM and subsequently observed by confocal microscopy, λ_exc_ = 405 nm, λ_em_ = 420–550 nm. Under the same instrument conditions, no auto-fluorescence of non-treated cells was detected.

Since DAE-NH-Py showed a distinct cytotoxic effect when irradiated, we took advantage of its fluorescence to study its cellular uptake and intracellular distribution in A549 cells by confocal microscopy. DAE-NH-Py at 10 µM efficiently entered live cells within 90 min incubation at 37 °C, 5% CO_2_ and non-selectively distributed within the cytoplasm (Figure 14).

## 3. Conclusions

Here, we described the synthesis of three novel diarylethene (DAE) photoswitches and fully characterized their spectroscopic and photochemical properties in MeOH and aqueous solution. All derivatives showed excellent photo-switching property of central DAE core in MeOH, comparable to previously published close analogues. [13] Intriguingly, photo-induced cyclisation kinetics of DAE showed to be concentration-dependent, for *c*(DAE-dye) > mM significantly slowing down, which was attributed to aromatic stacking interactions of DAE at such high concentrations: property not previously reported for DAE analogues. Moreover, in aqueous solution only -NH_2_ and NH-Boc derivatives showed fast and efficient DAE photo-switching between open and closed form comparable to MeOH solution, whereas DAE of bis-pyrene derivative (DAE-NH-Py) was readily photo-chemically closed but could not be reversely photo-chemically opened. Intriguingly, open DAE-NH-Py was characterized by strong pyrene-aggregate emission at 480 nm, which was upon photo-cyclisation completely quenched to give rise to weak pyrene emission at 400 nm, suggesting stacking of pyrene to another aryl (DAE). Such photochemical irreversibility in water was attributed to strong intramolecular aromatic stacking interactions of pyrenes with cyclic (closed) DAE unit, which prevented the photo-induced opening of DAE. Such solvent-dependent impact on DAE photochemistry, actually controlled by intramolecular aromatic stacking interactions, was to the best of our knowledge not reported until now and could be used in multi-dynamic control of the photo-switching process.

Further, to explore the bio-applicability of novel compounds, we studied their interactions with the most common biomacromolecular targets for small molecules: ds-DNA, ds-RNA and biologically most abundant protein BSA. Referent DAE-NH_2_ did not interact significantly with any of the studied DNA/RNA. Only open DAE-NH-Py showed measurable interactions with ds-DNA and ds-RNA with affinity in the micromolar range and fluorimetric selectivity for only AT(U) polynucleotides, attributed to deep insertion in respective grooves. For GC-DNA open DAE-NH-Py fluorimetric change was negligible due to blocked minor groove. Oppositely, closed DAE-NH-Py analogue did not interact with DNA/RNA, likely due to intramolecularly stacked conformation, too large for insertion into DNA/RNA groove. Very intriguingly, both, open and closed DAE-NH-Py interacted with BSA but in very different modes. More flexible open DAE-NH-Py was bound at three different BSA binding sites with very high cumulative affinity, while self-stacked closed DAE-NH-Py binds to only one.

Next, for the fluorescent DAE-NH-Py efficient cellular uptake in human tumor cells was demonstrated, whereby dye was localized in the cytoplasm, nonetheless we determined that studied compounds are non-toxic to human cell lines. However, when DAE-NH-Py treated cells were exposed for only 30 min to UV-light (315–400 nm), a strong cytotoxic effect was observed, attributed to pyrene ability to produce singlet oxygen-related species under irradiation.

Thus, particularly DAE-NH-Py, combining fluorescent emission with photo-induced bioactivity, can be considered as a promising lead compound for the development of photo-induced theranostic agents [5,28], particularly in PDT therapy under two-photon absorption (PTA) [29] conditions.

## 4. Materials and Methods

### 4.1. General

Solvents were distilled from appropriate drying agents shortly before use. TLC was carried out on TLC Silica gel 60 F254 Plastic sheets and preparative thin layer (2 mm) chromatography was done on Merck 60 F254 plates (Merck KGaA, Darmstadt, Germany). NMR spectra were recorded on AV600 and AV300 MHz spectrometers (Bruker BioSpin GmbH, Rheinstetten, Germany), operating at 600.13 or 300.13 MHz for ^1^H nuclei and at 150.92 MHz or 75.46 MHz for ^13^C, using DMSO-*d*_6_ (δ_H_: 2.50 ppm, δ_C_: 39.52 ppm) or CDCl_3_ (δ_H_: 7.26 ppm, δ_C_: 77.16 ppm) as the internal standard. Mass spectrometry was performed on an Agilent 6410 Triple Quad mass spectrometer (Agilent Technologies, Santa Clara, CA, USA). High-resolution mass spectra (HRMS) were obtained using a Q-TOF2 hybrid quadrupole time-of-flight mass spectrometer (Micromass, Cary, NC, USA).

### 4.2. Synthesis

Compound S (200.0 mg, 0.57 mmol) was dissolved in dichloromethane in the three-necked flask (8.0 mL) under an argon atmosphere. The solution was cooled in an ice bath and N-methylmorpholine (127.0 μL, 1.16 mmol) and 2-chloro-4,6-dimethoxy-1,3,5-triazine (200.0 mg, 1.14 mmol) were added.

**Figure 15:**
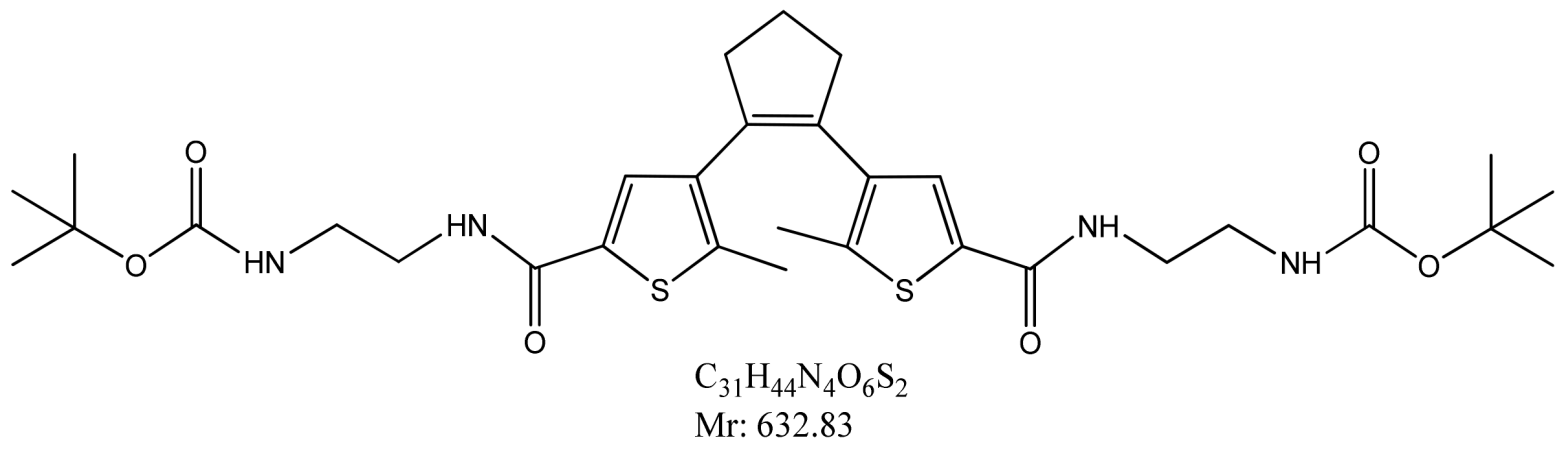
di-tert-butyl(((4,4’-(cyclopent-1-ene-1,2-diyl)bis(5-methylthiophene-4,2-diyl-2-carbonyl))bis(azanediyl))bis(ethane-2,1-diyl))dicarbamate (DAE-NH-Boc).

The reaction mixture was stirred for two hours in an ice bath, then the second portion of N-methylmorpholine (127.0 μL, 1.16 mmol) and Boc-ethylenediamine 7 (68.0 μL, 1.15 mmol) was added. After stirring for an additional 1 h in an ice bath, the reaction mixture was allowed to warm to room temperature overnight. The next day it was transferred to an extraction funnel with dichloromethane (12.0 mL) and washed twice with 1M HCl (13.0 mL), brine (31.0 mL), sodium bicarbonate (31.0 mL) and distilled water (41.0 mL). The organic layer was then dried over anhydrous Na_2_SO_4_. After evaporation of the solvent, the desired product DAE-NH-Boc (Figure 15) was isolated by preparative thin-layer chromatography in a solvent system of 9:1 dichloromethane-methanol (clear oil, 49.0 mg, 14%).

^1^H NMR (300 MHz, CDCl_3_) δ 7.23 (s, 2H, 2CH, DAE), 6.85 (br s, 2H, 2NH), 5.05 (br s, 2H, 2NH), 3.48 (q, *J* = 10.6, 5.0 Hz, 4H, 2CH_2_), 3.35 (q, *J* = 4.6 Hz, 4H, 2CH_2_), 2.76 (t, *J* = 7.4 Hz, 4H, 2CH_2_, DAE), 2.10–1.98 (m, *J* = 14.7, 7.5 Hz, 2H, DAE), 1.89 (s, 6H, 2CH_3_, DAE), 1.43 (s, 18H, BOC) ppm. ^13^C NMR (75 MHz, CDCl_3_, 25 °C, TMS) δ 162.57 (qC, CONHR), 157.55 (Qc, CO_2_NH), 140.15 (Qc, DAE), 136.41 (Qc, DAE), 134.90 (Qc, DAE), 134.52 (Qc, DAE), 129.44 (2CH, DAE), 80.07 (qC, BOC), 41.87 (2CH_2_), 40.20 (2CH_2_), 38.66 (CH_2_, DAE), 28.52 (6CH_3_, BOC), 23.06 (2CH_2_, DAE), 14.80 (2CH_3_, DAE) ppm. HRMS (MALDI-TOF/TOF)): calcd for C_31_H_44_N_4_O_6_S_2_Na^+^ [M–Na]^+^: 655.2599; found: 655.3011.

**Figure 16:**
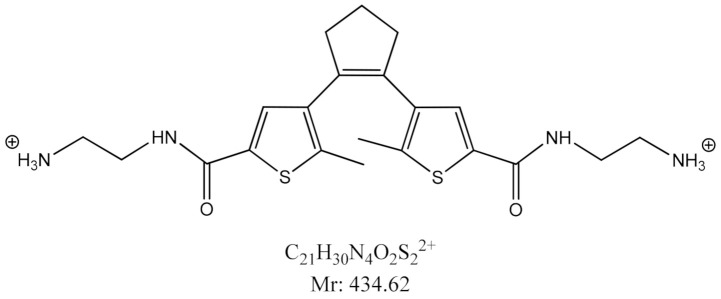
2,2′-((4,4′-(cyclopent-1-ene-1,2-diyl)bis(5-methylthiophene-2,2′-carbonyl))bis(azanediyl))diethanaminium (DAE-NH_2_).

In a round-bottomed flask, DAE-NH-Boc (35.0 mg, 0.06 mmol) was dissolved in dichloromethane (4.0 mL) after which the solution was cooled in an ice bath. Trifluoroacetic acid (4.0 mL, 52.24 mmol) was then slowly added dropwise to the cooled reaction mixture with stirring over half an hour. This was followed by stirring at room temperature for about 2 h until the complete disappearance of the starting compound was carried out by thin-layer chromatography in a 9:1 dichloromethane-methanol system. Product DAE-NH_2_ (Figure 16) was obtained as a white solid (30.0 mg, 100%).

^1^H NMR (600 MHz, MeOD) δ 7.53 (s, 2H, 2CH, DAE), 3.63 (t, *J* = 5.9 Hz, 4H, 2CH_2_), 3.17 (t, *J* = 5.9 Hz, 4H, 2CH_2_), 2.87 (t, *J* = 7.5 Hz, 4H, 2CH_2_, DAE), 2.18–2.12 (m, 2H, DAE), 1.96 (s, 6H, 2CH_3_, DAE) ppm. ^13^C NMR (151 MHz, MeOD, 25 °C, TMS) δ 165.24 (Qc, CONHR), 142.23 (qC, DAE), 138.01 (Qc, DAE), 136.18 (Qc, DAE), 135.26 (Qc, DAE), 131.30 (2CH, DAE), 41.07 (2CH_2_), 39.64 (2CH_2_), 38.67 (CH_2_, DAE), 23.50 (2CH_2_, DAE), 14.46 (2CH_3,_ DAE) ppm. HRMS (MALDI-TOF/TOF)): calcd for C21H30N4O2S22^+^[M-H]^+^: 433.1732; found: 433.1743.

**Figure 17:**
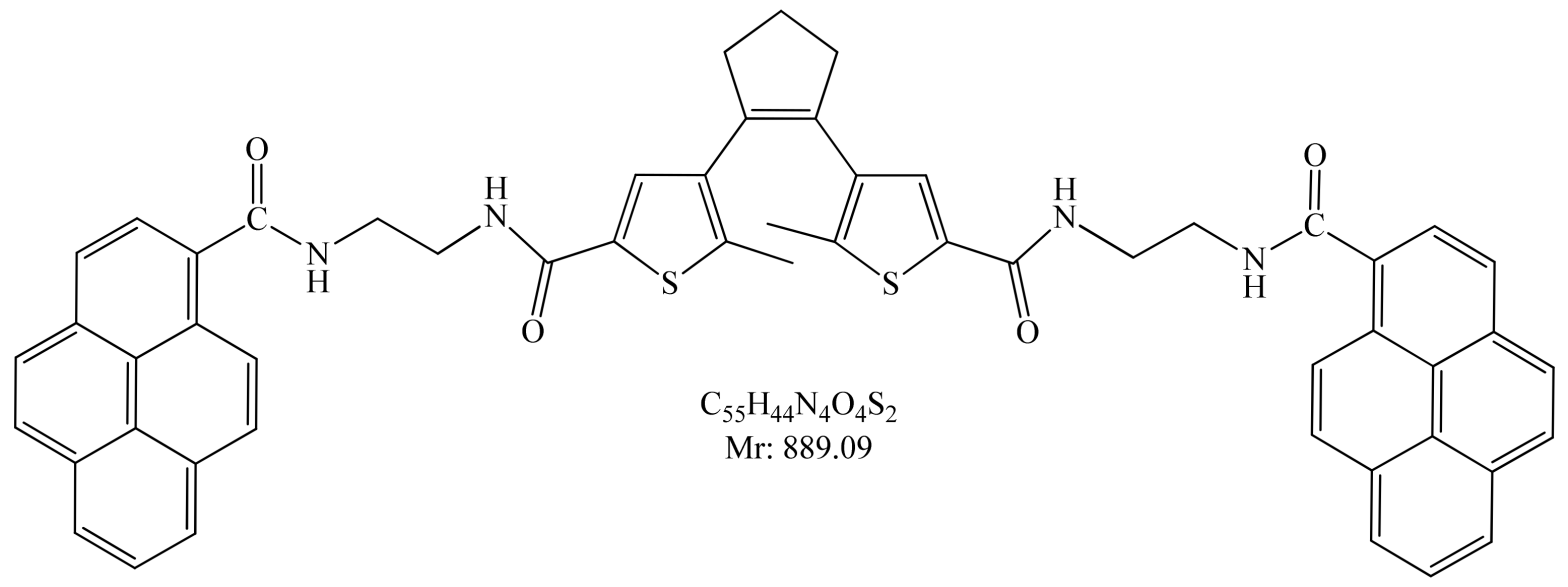
4,4′-(cyclopent-1-ene-1,2-diyl)bis(5-methyl-N-(2-(pyrene-1-carboxamido)ethyl)thiophene-2-carboxamide) (DAE-NH-Py).

Compound S (54.4 mg, 0.16 mmol) was dissolved in dichloromethane (2.0 mL) under an argon atmosphere. The reaction mixture was cooled in an ice bath and N-methylmorpholine (34.8 μL, 0.32 mmol) and 2-chloro-4,6-dimethoxy-1,3,5-triazine (54.4 mg, 0.31 mmol) were added. The reaction mixture was stirred in an ice bath for 2 h, then the second portion of N-methylmorpholine (34.8 μL, 0.32 mmol) was added. Compound 10 (90.0 mg, 0.31 mmol) was suspended in dichloromethane (4.0 mL) with the addition of dimethylformamide (300.0 μL) followed by gradual dropwise addition to the reaction mixture. The reaction mixture was continued to stir at room temperature for 48 h, after which it was transferred to an extraction funnel with dichloromethane (3.3 mL). It was washed twice with 1M HCl (5.0 mL), brine (5.0 mL), sodium bicarbonate (7.0 mL) and distilled water (15.0 mL). The organic layer was dried over anhydrous Na_2_SO_4_. Evaporation of the solvent gave a brown oil from which the desired product DAE-NH-Py (Figure 17) was isolated by preparative thin-layer chromatography in a 9:1 dichloromethane-methanol solvent system (white solid, 7.0 mg, 5%).

^1^H NMR (600 MHz, DMSO) δ 8.77 (t, 2H, 2NH), 8.54 (t, 2H, 2NH), 8.51 (d, *J* = 9.2 Hz, 2H, Py), 8.33 (dd, *J* = 17.5, 7.7 Hz, 6H, Py), 8.27–8.10 (m, 10H, Py), 7.59 (s, 2H, 2CH, DAE), 3.57 (q, 4H, 2CH_2_), 3.52 (q, 4H, 2CH_2_), 2.78 (t, 4H, 2CH_2_, DAE), 2.06–1.99 (m, 2H, CH_2_, DAE), 1.82 (s, 6H, 2CH_3_, DAE) ppm. ^13^C NMR (75 MHz, DMSO, 25 °C, TMS) δ 169.11 (CONHPy), 161.27 (CONHR), 138.86 (qC, DAE), 136.05 (qC, DAE), 135.81 (qC, DAE), 134.11 (qC, DAE), 131.92 (qC, Py), 131.60 (qC, Py), 130.73 (qC, Py), 130.19 (qC, Py), 128.92 (2CH, DAE), 128.30 (CH, Py), 128.00 (CH, Py), 127.80 (qC, Py), 127.23 (CH, Py), 126.60 (CH, Py), 125.81 (CH, Py), 125.57 (CH, Py), 125.34 (CH, Py), 124.83 (CH, Py), 124.40 (CH, Py), 123.78 (qC, Py), 123.64 (qC, Py), 38.38 (2CH_2_, DAE), 22.30 (CH_2_, DAE), 14.24 (2CH_3_, DAE). HRMS (MALDI-TOF/TOF)): calcd for C_55_H_44_N_4_O_4_S_2_Na^+^ [M–Na]^+^: 911.2702; found: 911.2674.

### 4.3. Study of DNA/RNA Interactions

All measurements were performed in aqueous buffer solution (pH = 7.0, *I* = 0.05 M, sodium cacodylate buffer). The UV-Vis spectra were recorded on a Varian Cary 100 Bio spectrometer, fluorescence spectra were recorded on a Varian Cary Eclipse fluorimeter and CD spectra were recorded on JASCO J815 spectropolarimeter at 25.0 °C using appropriate quartz cuvettes (path length: 1 cm).

Polynucleotides were purchased as noted: poly dAdT—poly dAdT, poly dGdC—poly dGdC, poly A—poly U, (Sigma), *calf thymus* (ct)—DNA (Aldrich) and dissolved in sodium cacodylate buffer, *I* = 0.05 M, pH = 7.0. The ct-DNA was additionally sonicated and filtered through a 0.45 mm filter to obtain mostly short (ca. 100 base pairs) rod-like B-helical DNA fragments [45]. Polynucleotide concentration was determined spectroscopically [46] as the concentration of phosphates (corresponds to *c*(nucleobase)).

Circular dichroism (CD) spectra were recorded on JASCO J-815 spectropolarimeter at room temperature using 1 cm path quartz cuvettes with a scanning speed of 200 nm/min (an average of three accumulations). A buffer background was subtracted from each spectrum. CD experiments were performed by adding portions of the compound stock solution into the solution of the polynucleotide (*c* = 2 × 10^−5^ M).

Thermal melting curves for ds-DNA, ds-RNA and their complexes with studied compounds were determined as previously described [39] by following the absorption change at 260 nm as a function of temperature. The absorbance of the ligands was subtracted from every curve and the absorbance scale was normalized. *T_m_* values are the midpoints of the transition curves determined from the maximum of the first derivative and checked graphically by the tangent method. The ∆*T_m_* values were calculated by subtracting *T_m_* of the free nucleic acid from *T_m_* of the complex. Every ∆*T_m_* value here reported was the average of at least two measurements. The error in ∆*T_m_* is ± 0.5 °C.

### 4.4. Biology

Cells: A549 (human lung carcinoma; ATCC CCL-185) were obtained from the ATCC Cell Biology Collection and were cultured according to the manufacturer’s instructions. Cells were grown in Dulbecco Modified Eagle’s Medium (DMEM, Sigma Aldrich, St. Louis, MO, USA) supplemented with 10% of fetal bovine serum (FBS, Sigma Aldrich, USA) at 37 °C and 5% CO_2_ in a humified atmosphere. Three biological replicas have been performed for all experiments.

Cytotoxicity assay; MTT: Studied compounds were dissolved in the appropriate volume of dimethyl sulfoxide solution (DMSO) under sterile conditions, to obtain the stock of 10 mM solution and kept in the dark at +4 °C. Before each assay, a new fresh working solution has been prepared from the stock solution by diluting it with DMEM. Cells were seeded on 96 well plate in the concentration of 7 × 10^3^ cells/well in 100 μL of DMEM (10% FBS) and left in the incubator overnight (37 °C, 5% CO_2_). The next day, 100 μL of the working solution was added to the wells, thus the final concentration of tested compounds was obtained in the total volume of 200 μL/well. All conditions were tested in quadruplicates. Cells treated with the same dilutions of DMSO represented control, while cells treated only with DMEM (10% FBS) represented negative control. The plate was then incubated for the next 72 h (37 °C, 5% CO_2_). After the incubation, the medium was removed and the 40 μL of MTT solution was added to each well. The plate was incubated in the cell incubator for 3 h, allowing the formazan crystals to form. After 3 h, 170 μL of DMSO was added to each well and put on a shaker for 20 min, allowing crystals to dissolve. The absorbance of the MTT-formazan product was measured with a microplate reader at 600 nm. Absorbance value directly correlates with a cell survival.

For UV-A irradiation experiments cell cultures plates prepared as above were treated with studied compounds and irradiated in a Luzchem reactor with eight overhead-mounted LZC-UVA lamps (in total 64 W, 315–400 nm, Dose 50.6 mw·m^−2^), ~18 cm lamp to cell-plate distance; at 90 min, 24 h and 48 h after treatment. Exposure to light was 30 min per day.

Live cell imaging: Live imaging of the cells treated with compounds was performed on the A549 cell line. Cells were seeded in Ibidi imaging cell chambers (Ibidi^®^, Martinsried, Germany) in 500 μL of the medium, with the concentration of 5 × 10^4^ cells/well and left in the cell incubator for 48 h (37 °C, 5% CO_2_). After two days, cells were treated with 10 μM solution of a tested compound and left in the cell incubator for 90 min to allow the compound to enter the cells. After incubation, the medium was replaced with 500 μL of fresh DMEM and cells were immediately observed by Leica SP8 X confocal microscope (Leica Microsystems, Wetzlar/Mannheim, Germany).

## Figures and Tables

**Table 1:** Electronic absorption data of DAE-NH-Boc, DAE-NH_2_ and DAE-NH-Py (open and closed form).

Compound	λ (MeOH) /nm	ε (MeOH) /mmol^−1^ cm^2^	λ (Buffer) /nm	ε (Buffer) /mmol^−1^ cm^2^
DAE-NH-Boc	261	95,211	266	22,423
DAE-NH_2_	-	-	264	17,588
DAE-NH-Py (open)	275	43,206	280	29,577
	341	27,724	348	17,637
^a^ DAE-NH-Py (closed)	275	46,910	280	36,254
			347	26,792
			530	4252

^a^ Prepared by photochemical reaction in MeOH as described under Section 2.3.1, MeOH subsequently evaporated and “DAE-NH-Py closed form” re-dissolved in buffer (<1%DMSO).

**Table 2:** ^a^ ∆T_m_-Values (°C) for different ratios ^b^ r of DAE-NH_2_ and DAE-NH-Py added to the polynucleotide.

Compound	^b^ r	ct-DNA	poly A—poly U
DAE-NH_2_ (open form)	0.3	−1.0	−0.7
DAE-NH-Py (open form)	0.1		0.0
	0.2		0.0/26.1
	0.3	−0.7	0/23.4
DAE-NH-Py (closed form)	0.3	−1.9	0.3

^a^ ∆T_m_-Value error ± 0.5 °C; ^b^ r = [compound]/[polynucleotide].

**Table 3:** Binding constants and emission changes (^a^ log*Ks* (M^−1^)/^b^ ΔInt(%)) of open DAE-NH-Py complexes with ds-polynucleotides calculated by processing fluorimetric titrations.

ct-DNA	AT-DNA	GC-DNA	AU-RNA
5.6/26	6.3/11	^c^	6.1/22

^a^ Processing of titration data by means of Scatchard Equation [42] gave values of ratio n[bound dye]/[polynucleotide] = 0.1–0.2, for easier comparison all log*Ks* values were re-calculated for fixed n = 0.2. Correlation coefficients were > 0.99 for all calculated log*K_s_*. ^b^ ΔInt = [(Int(DAE-NH-Py)-Int_complex_)/Int(DAE-NH-Py)] × 100; ^c^ Too small changes for accurate processing by Scatchard eq.

## Data Availability

All data is contained within this manuscript and the Supplementary Materials file.

## Supplementary Materials

The following are available online at https://www.mdpi.com/article/10.3390/ijms22094916/s1, additional data about synthetic procedures, structural properties of studied DNA and RNA; physico-chemical properties of studied compounds aqueous solutions, additional experimental data (fluorimetric and CD titrations, thermal denaturation experiments) of interactions with biomacromolecules.
