# Supplementary material for: Bis-Pyrene Photo-Switch Open- and Closed-Form Differently Bind to ds-DNA, ds-RNA and Serum Albumin and Reveal Light-Induced Bioactivity
Source: Int J Mol Sci. 2021 May 6;22(9):4916. doi: 10.3390/ijms22094916 (PMC8125568; doi:10.3390/ijms22094916)
Supplement: Supplementary file 1 [file ijms-22-04916-s001.zip › ijms-1194168-supplementary.pdf]

**Supporting Information:**

# Bis-pyrene photo-switch open- and closed-form differently bind to ds-DNA, ds-RNA and serum albumin and reveal light-induced bioactivity

**Iva Orehovec[1], Marija Matković[1], Isabela Pehar[2], Dragomira Majhen[2], Ivo Piantanida[1,*]**

[1] Division of Organic Chemistry and Biochemistry, Ruđer Bošković Institute, Bijenička Cesta 54, 10000 Zagreb, Croatia; Iva.Orehovec@irb.hr (I.O.), Marija.Matkovic@irb.hr (M.M.)
[2] Division of Molecular Biology, Ruđer Bošković Institute, Bijenička Cesta 54, 10000 Zagreb, Croatia; Isabela.Pehar@irb.hr (Is.P.); Dragomira.Majhen@irb.hr (D.M.)
* Correspondence: pianta@irb.hr; Tel.: +385-1-4571-326 (Iv.P.)

Synthesis:

The starting compound 2-chloro-5-methyl-thiophene **3** for the synthesis of diarylethene core **S** was prepared by chlorination[1,2] of 2-methylthiophene **1** at the 5-position with N-chlorosuccinimide (NCS) **2** in a mixture of acetic acid and benzene. After that, the obtained product **3** was subjected to a Friedel-Crafts reaction[3] with aluminum (III) chloride ($AlCl_3$) and glutaryl chloride **4** in nitromethane at 0 °C. The obtained 1,5-diketone **5** was then used in a McMurry reaction[4] with titanium tetrachloride (TiCl4) and zinc (Zn) as a reduction agent in tetrahydrofuran (THF) to obtain chlorinated dithienylcyclopentene **6**. After that, diacid dithienylcyclopentene switch **7** was obtained in dry THF with n-Bu-Li by introducing solid $CO_2$ into the reaction. Desired compound **S** was obtained in 91.06 % yield and used without further purification.

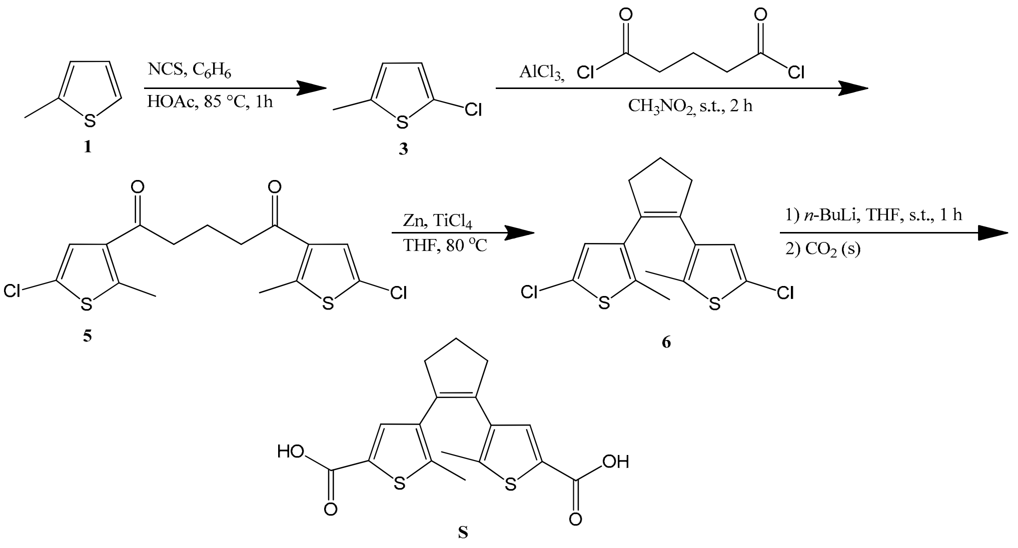


Scheme S1. Synthesis of 4,4'-(cyclopent-1-ene-1,2-diyl)bis(5-methylthiophene-2-carboxylic acid) **S**

**2-chloro-5-methylthiophene (3)**

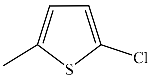

Benzene (100.0 mL), acetic acid, methylthiophene **1** (25.0 mL) and N-chlorosuccinimide **2** (37.5 g) were added to the reaction flask and stirred vigorously at room temperature for 30 min followed by reflux at 85 °C for 1 h. The cooled mixture was transferred to an extraction funnel with 3 M sodium hydroxide solution (75.0 mL). The alkaline portion was drained and the remainder of the mixture was washed 2 more times with 3 M sodium hydroxide solution (75.0 mL) and finally with distilled water (30.0 mL). The organic layer was then dried over anhydrous $Na_2SO_4$. After evaporation of the solvent, the product was isolated from the reaction mixture by vacuum distillation using a Liebieg condenser connected to a rotavapor (25 millibars, 130-160 °C). Product **3** was obtained as a clear oil (11.9 mg, 35 %).

**1,5-bis (5-chloro-2-methylthiophen-3-yl) pentane-1,5-dione (5)**

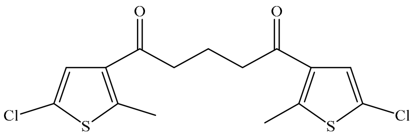

Compound **3** (5.0 mg) was dissolved in nitromethane (39.0 mL) in a three-necked reaction flask under an argon atmosphere and glutaryl chloride **4** (2.4 mL) was added. The reaction mixture was then cooled in an ice bath and aluminum (III) chloride (6.0 g) was gradually added over half an hour followed by stirring at room temperature for 2 h. The reaction was quenched by the addition of pre-cooled distilled water (1.0 mL) after which was transferred to an extraction funnel and extracted three times with diethyl ether (30.0 mL). The combined ether phases were washed with distilled water (30.0 mL) and dried over anhydrous $Na_2SO_4$. Evaporation of the solvent gave brown product **5** as an oil (7.3 g, 51 %). The resulting product **5** was used without further purification.

**1,2-bis (5-chloro-2-methylthiophen-3-yl) cyclopent-1-ene (6)**

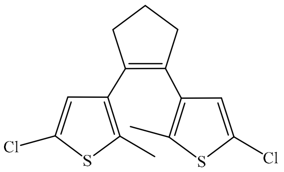


Zinc was activated according to the procedure from the literature.[5] Activated zinc (10.0 g) was then added to the reaction flask with tetrahydrofuran (400.0 mL). The mixture was then cooled in an ice bath and stirred under an argon atmosphere after which titanium tetrachloride (22.5 mL) was added. This was followed by heating the reaction mixture to 85 °C for one hour, after which reaction turned dark blue. Mixture was then cooled again in an ice bath and compound **5** dissolved in tetrahydrofuran (105.0 mL) was added dropwise over half an hour. The reaction mixture was then reheated to 85-87 °C for another 72 hours. After cooling to room temperature, the reaction was quenched by the addition of 1M $NH_4C_l$ (92.0 mL). The tetrahydrofuran was evaporated from the reaction flask by rotavapor at 40 °C. The reaction mixture was then transferred to an extraction funnel and extracted 4 times with diethyl ether (500.0 mL). The combined ether phases were washed with distilled water (250.0 mL) and dried over $Na_2SO_4$. After evaporation, the resulting crude mixture was dissolved in petroleum ether and applied to sinter filled with silica gel. The sinter was washed with petroleum ether until all product **6** was isolated from it as a clear oil (5.7 g, 51 %).

**4,4'-(cyclopent-1-ene-1,2-diyl)bis(5-methylthiophene-2-carboxylic acid) (S)**

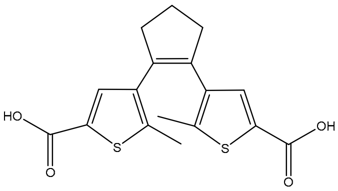


Compound **6** was dissolved in tetrahydrofuran (7.0 ml) and added through the septum to a reaction flask filled with argon. After stirring at room temperature for a few minutes, n-Bu-Li (1.4 ml) was added dropwise to the solution over 10 minutes, during which time the reaction mixture began to thicken and take on a dark color. After stirring for one hour at room temperature, a suspension of ocher color was obtained. Solid $CO_2$ was then added

to the reaction flask in excess and the reaction mixture was continued to stir overnight in an oil bath at 25 °C. The next day, distilled water (7.0 ml) was added to a thick ocher suspension. Tetrahydrofuran was then evaporated from the reaction flask under reduced pressure at 40 °C. The reaction mixture was transferred to the extraction funnel and washed with diethyl ether (10.0 ml). The aqueous phase was acidified with 30 % hydrochloric acid from pH 7 to pH 1 after which everything was transferred to the extraction funnel again and extracted three times with dichloromethane (10.0 ml). The ether phases were washed with distilled water (25.0 ml) and dried over $Na_2SO_4$. Evaporation gave product **S** as a light brown color (214.0 mg, 91 %), which was not required for further purification.

**Di-tert-butyl(((4,4'-(cyclopent-1-ene-1,2-diyl)bis(5-methylthiophene-4,2-diyl-2-carbonyl))bis(azanediyl))bis(ethane-2,1-diyl))dicarbamate (DAE-NH-Boc)**:

In another step we decided to extend diarylethene core **S** with N-Boc-ethylenediamine **7** on both sides (Scheme S2). To obtain this, **DAE-NH-Boc** was prepared by coupling method well known in peptide chemistry[7], under mild conditions, starting from compound **S.** The carboxylic acid was first deprotonated by N-methylmorpholine (NMM) and subsequently activated by 2-chloro-4,6-dimethoxytriazine. This was followed by reaction of the activated ester with the corresponding amine **7**. The protected amide-functionalized switch **DAE-NH-Boc** was isolated in 14 % yield.

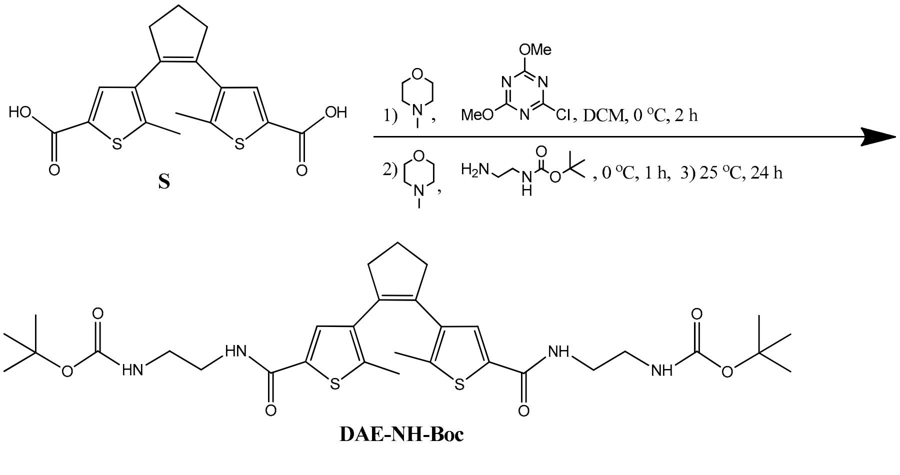


Scheme S2. Synthesis of **DAE-NH-Boc**

**2,2'-((4,4'-(cyclopent-1-ene-1,2-diyl)bis(5-methylthiophene-2,2'-carbonyl))bis(azanediyl))diethanaminium (DAE-NH2):**

Diamine derivate **DAE-NH2** was obtained by Boc-deprotection of **DAE-NH-Boc**, according to the literature procedure.[11] Deprotection reaction with TFA / $CH_2Cl_2$ proceeded smoothly and **DAE-NH2** was obtained in quantitative yield (Scheme S3).

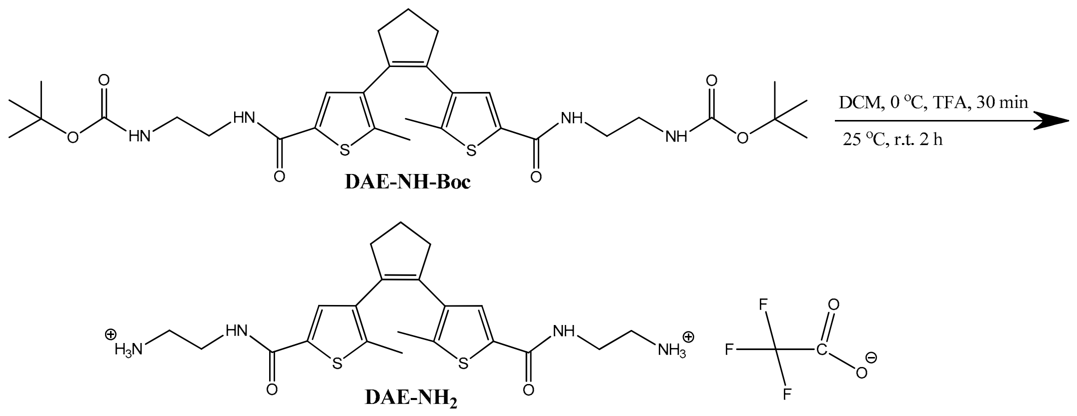


Scheme S3. Synthesis of **DAE-NH2**

**N-(2-aminoethyl)pyrene-1-carboxamide (10):**

First attempt to prepare desired pyrene derivative **DAE-NH-PY** by simple coupling of **DAE-NH2** with 1-aminopyrene was unsuccessful due to complete degradation of the product during purification. Therefore, we decided to synthesize product **10** in 3 steps (Scheme S4), starting from N-Boc-ethylenediamine **7** which was coupled with 1-pyrenecarboxylic acid **8** by standard coupling reaction[6] using HCTU coupling reagent and triethylamine (TEA) in acetonitrile. Desired conjugate **9** was obtained in 63 % yield. In another step Boc-deprotection was performed on product **9** with TFA / $CH_2Cl_2$ after which obtained product was subsequently neutralized with potassium hydroxide (NaOH) and isolated as desired product **10** in quantitative yield. In the last step, pyrene derivate **DAE-NH-PY** was synthesized (Scheme S5) by the same coupling method already described[7] and isolated in poor 5 % yield.

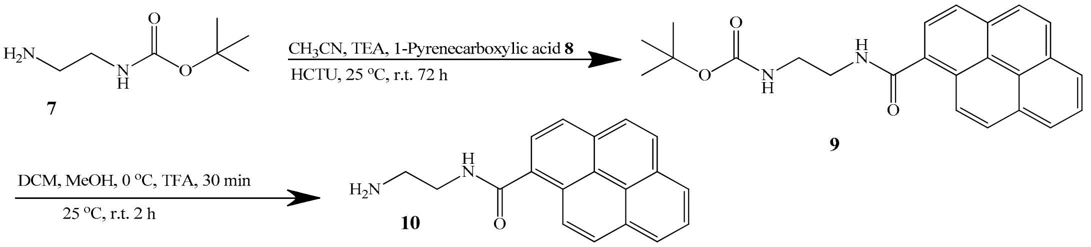


Scheme S4. Synthesis of N-(2-aminoethyl)pyrene-1-carboxamide **10**

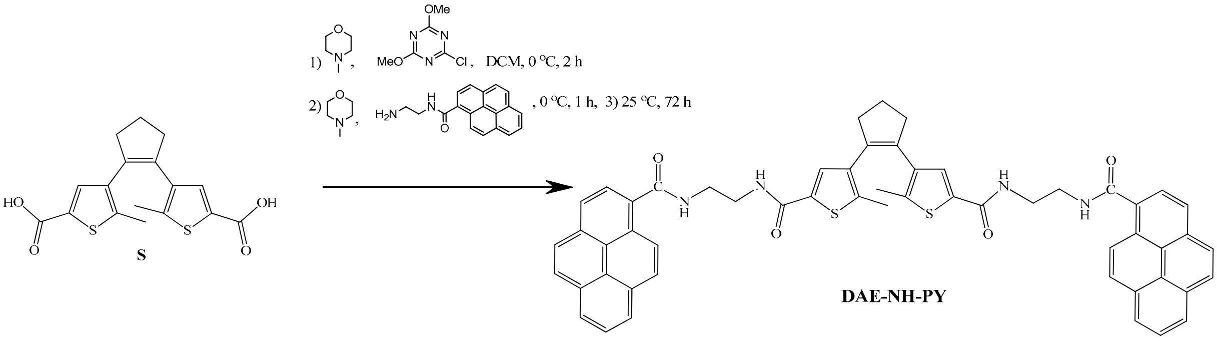


Scheme S5. Synthesis of pyrene derivate **DAE-NH-PY**

**Tert-butyl (2-(pyrene-1-carboxamido)ethyl)carbamate (9)**

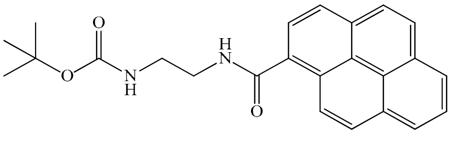

$C_{24}H_{24}N_2O_3$
Mr: 388.46

In a round-bottomed flask, N-Boc-ethylenediamine **7** (204.0 mg, 1.27 mmol) was dissolved in acetonitrile (10.0 mL) after which triethylamine (340.0 µL, 2.44 mmol) and 1-Pyrenecarboxylic acid **8** (300.0 mg, 1.22 mmol) were added. HCTU (526.0 mg, 1.27 mmol) was then dissolved in freshly distilled acetonitrile (2.0 mL) and gradually added dropwise to the yellow mixture with stirring at room temperature. The resulting white suspension was then continued to stir under an argon atmosphere and at room temperature for 72 h. The reaction was quenched by the addition of saturated aqueous sodium chloride solution (34.0 mL). The aqueous layer was extracted three times with ethyl acetate (25.0 mL). The combined organic layers were washed with 2M hydrochloric

acid solution (3.0 mL), distilled water (3.0 mL), 5% sodium bicarbonate solution (20.0 mL) and distilled water (20.0 mL). The organic layer was then dried over anhydrous $Na_2SO_4$. After evaporation of the solvent, the desired product **9** was isolated from the reaction mixture by preparative thin layer chromatography in a 19: 1 dichloromethane-methanol solvent system (white solid, 190.0 mg, 63 %).

$^1$H NMR (300 MHz, $CDCl_3$) δ 8.60 (d, *J* = 9.3 Hz, 1H, Py), 8.21 (d, *J* = 7.6 Hz, 8H, Py), 8.14 – 7.99 (m, *J* = 9.0 Hz, 3.3, 6H, Py), 6.87 (s, 1H, NH), 5.11 (s, 1H, NH), 3.73 (q, *J* = 11.3 Hz, 5.5, 2H, $CH_2$), 3.49 (q, *J* = 11.3, 5.8 Hz, 2H, $CH_2$), 1.42 (s, 9H, $3CH_3$, BOC) ppm. $^{13}$C NMR (75 MHz, DMSO, 25 °C, TMS) δ 168.99 (qC, CONHR), 155.80 (qC, $CO_2NH$), 131.94 (qC, Py), 131.58 (qC, Py), 130.72 (qC, Py), 130.20 (qC, Py), 128.27 (CH, Py), 128.03 (CH, Py), 127.80 (qC, Py), 127.22 (CH, Py), 126.58 (CH, Py), 125.78 (CH, Py), 125.60 (CH, Py), 125.36 (CH, Py), 124.82 (CH, Py), 124.33 (CH, Py), 123.78 (qC, Py), 123.64 (qC, Py), 77.60 (qC, BOC), 39.71 ($CH_2$), 39.66 ($CH_2$), 28.28 ($3CH_3$) ppm. HRMS (MALDI-TOF/TOF)): calcd for $C_{24}H_24N_2O_3Na^+$ [M–Na]$^+$: 388.1787; found: 388.1783.

**N-(2-aminoethyl)pyrene-1-carboxamide (10)**

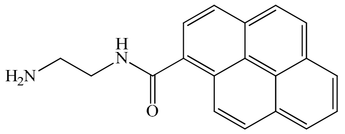


$C_{19}H_{16}N_2O$
Mr: 288.34

Compound **9** (120.0 mg, 0.31 mmol) was dissolved in a solvent mixture of dichloromethane-methanol-dioxane 44: 5: 1 (10.5 mL) after which the reaction mixture was cooled in an ice bath. Trifluoroacetic acid (10.5 mL, 137.00 mmol) was then slowly added dropwise to the cooled reaction mixture with stirring over half an hour. This was followed by stirring at room temperature for about 2 h, until thin layer chromatography in a 9: 1 dichloromethane-methanol system showed complete disappearance of the starting compound. The resulting white salt was suspended in distilled water (20.0 mL) and washed in an extraction funnel with dichloromethane (20.0 mL). The aqueous phase was neutralized with 2M potassium hydroxide (NaOH) solution to pH 10. The resulting amine

was extracted twice with dichloromethane (50.0 mL). After evaporation of the solvent, product **10** was obtained as a white solid (89.0 mg, 100 %).

$^{1}H$ NMR (600 MHz, DMSO) δ 8.64 (t, *J* = 5.3 Hz, 1H, NH), 8.51 (d, *J* = 9.2 Hz, 1H, Py), 8.37 – 8.32 (m, *J* = 14.0, 7.7 Hz, 3H, Py), 8.28 – 8.21 (m, *J* = 19.4 , 9.6 Hz, 3H, Py), 8.17 – 8.10 (m, 2H, Py), 3.43 (q, *J* = 12.2, 6.3 Hz, 2H, $CH_2$), 2.82 (t, *J* = 6.5 Hz, 2H, $CH_2$) ppm. $^{13}C$ NMR (151 MHz, DMSO, 25 °C, TMS) δ 168.97 (CONHR), 132.26 (qC, Py), 131.47 (qC, Py), 130.72 (qC, Py), 130.21 (qC, Py), 128.19 (CH, Py), 128.02 (CH, Py), 127.71 (qC, Py), 127.21 (CH, Py), 126.55 (CH, Py), 125.74 (CH, Py), 125.55 (CH, Py), 125.24 (CH, Py), 124.74 (CH, Py), 124.38 (CH, Py), 123.78 (qC, Py), 123.66 (qC, Py), 43.19 ($CH_2$), 41.42 ($CH_2$) ppm. HRMS (MALDI-TOF/TOF)): calcd for $C_{19}H_{17}N_2ONa^+$ [M–Na]$^+$: 289.1341; found: 289.1340.

**Study of interactions with DNA/RNA**

Polynucleotides were purchased as noted: poly A – poly U, poly dGdC – poly dGdC, poly dAdT – poly dAdT (Sigma), *calf thymus* (ct)-DNA (Aldrich) and dissolved in sodium cacodylate buffer, *I* = 0.05 M, pH=7.0. The ct-DNA was additionally sonicated and filtered through a 0.45 mm filter to obtain mostly short (ca. 100 base pairs) rod-like B-helical DNA fragments.[8] The polynucleotide concentration was determined spectroscopically, by molar absorptivity provided by manufacturer) as the concentration of phosphates (corresponds to *c*(nucleobase)).

Table S1. Groove widths and depths for selected nucleic acid conformations.[9,10]

| Structure type | Groove width [Å] | | Groove depth [Å] | |
|---|---|---|---|---|
| | major | minor | major | minor |
| [a]poly rA – poly rU | 3.8 | 10.9 | 13.5 | 2.8 |
| [b]poly dGdC – poly dGdC | 13.5 | 9.5 | 10.0 | 7.2 |
| [b]poly dAdT – poly dAdT | 11.2 | 6.3 | 8.5 | 7.5 |
| [b]ct-DNA | 11.2 | 6.3 | 8.5 | 7.5 |

[a] A-helical structure

[b] B- helical structure

In buffered solution (open form)

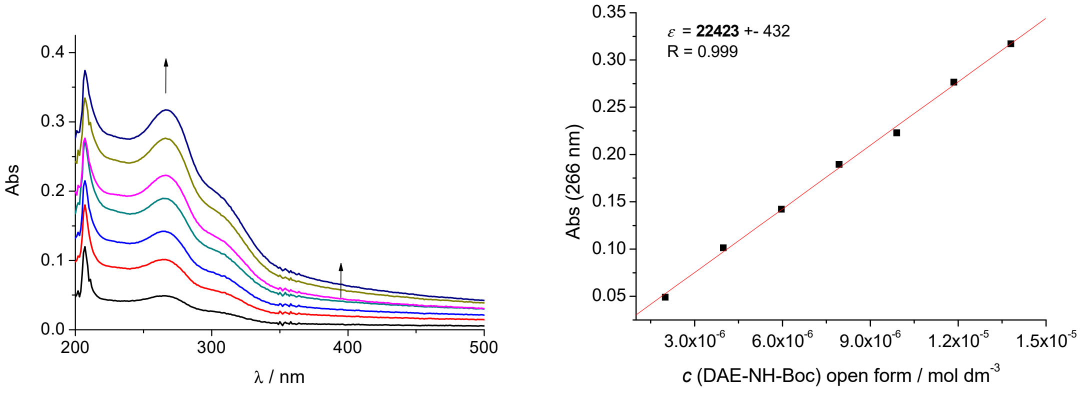


a) b)

**Figure S1. a)** Dependence of UV/Vis spectra on concentration of **DAE-NH-Boc (open form), b)** Dependence of UV/Vis spectra on concentration increase of **DAE-NH-Boc (open form,** $c$ = 1.4×10$^{-5}$ M) at pH 7.0, sodium cacodylate buffer, $I$ = 50 mM.

In MeOH (open form)

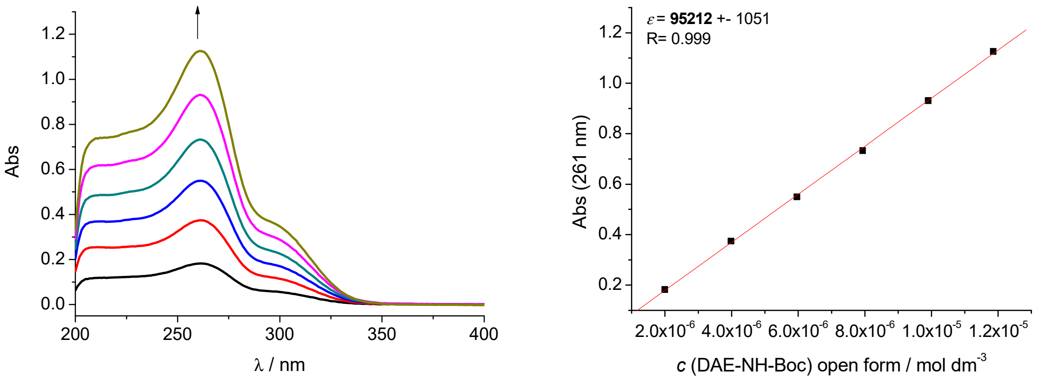


a) b)

**Figure S2. a)** Dependence of UV/Vis spectra on concentration of **DAE-NH-Boc (open form), b)** Dependence of UV/Vis spectra on concentration increase of **DAE-NH-Boc (open form**, $c$ = 1.2×10$^{-5}$ M) in MeOH.

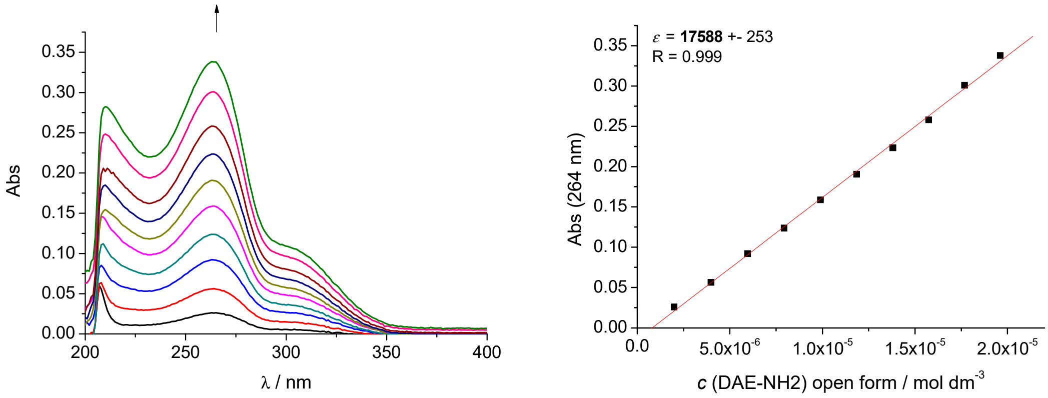


a) b)

**Figure S3. a)** Dependence of UV/Vis spectra on concentration of **DAE-NH$_2$ (open form), b)** Dependence of UV/Vis spectra on concentration increase of **DAE-NH$_2$ (open form**, *c* = 2.0×10$^{-5}$ M) at pH 7.0, sodium cacodylate buffer*, I* = 50 mM.

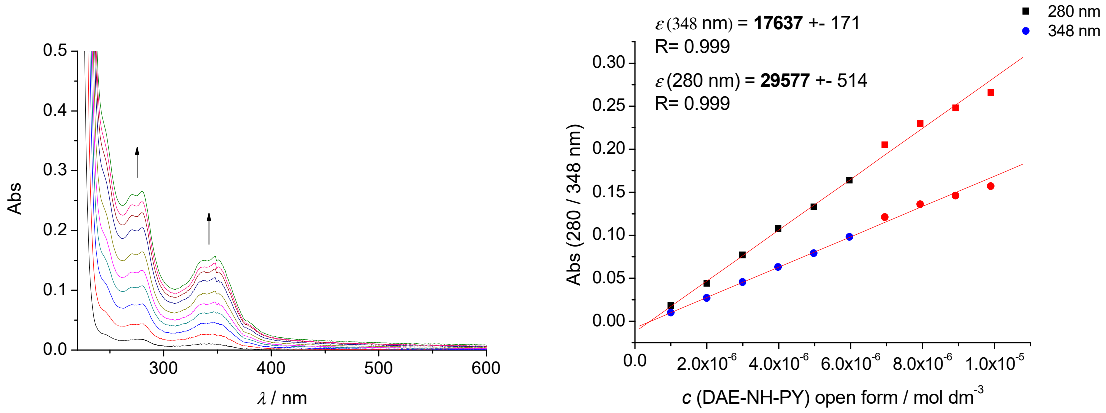


a) b)

**Figure S4. a)** Dependence of UV/Vis spectra on concentration of **DAE-NH-PY (open form), b)** Dependence of UV/Vis spectra on concentration increase of **DAE-NH-PY (open form)** (*c* = 9.9×10$^{-6}$ M) at pH 7.0, sodium cacodylate buffer*, I* = 50 mM / 1 % DMSO

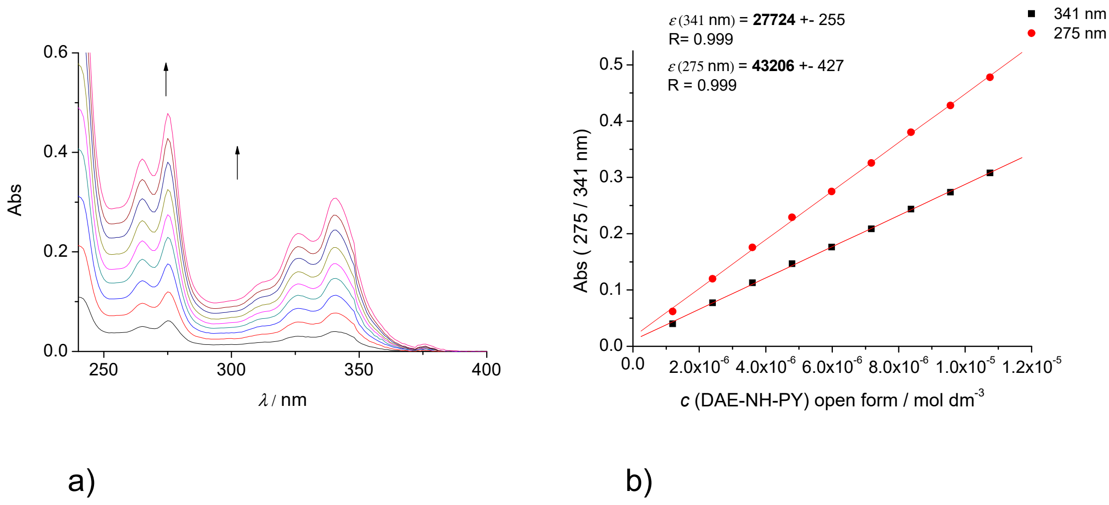


**Figure S5. a)** Dependence of UV/Vis spectra on concentration of **DAE-NH-PY (open form), b)** Dependence of UV/Vis spectra on concentration increase of **DAE-NH-PY (open form)** ($c$ = 1.0×10$^{-5}$ M) in MeOH / 0.5 % DMSO

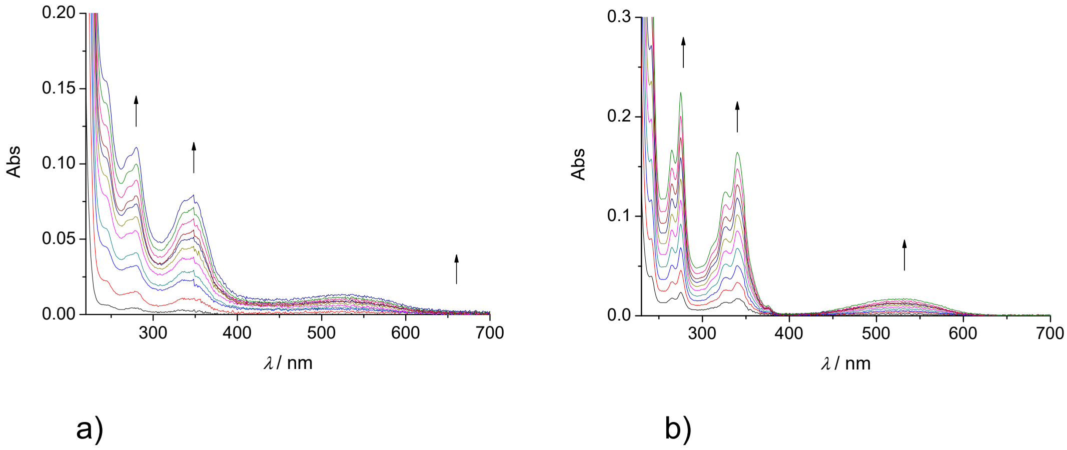


**Figure S6.** Dependence of UV/Vis spectra on concentration of **DAE-NH-PY (closed form)** in **a)** sodium cacodylate buffer (pH = 7.0, ***I*** = 50 mM) / 4 % MeOH / 0.2 % DMSO **b)** MeOH / 4 % DMSO

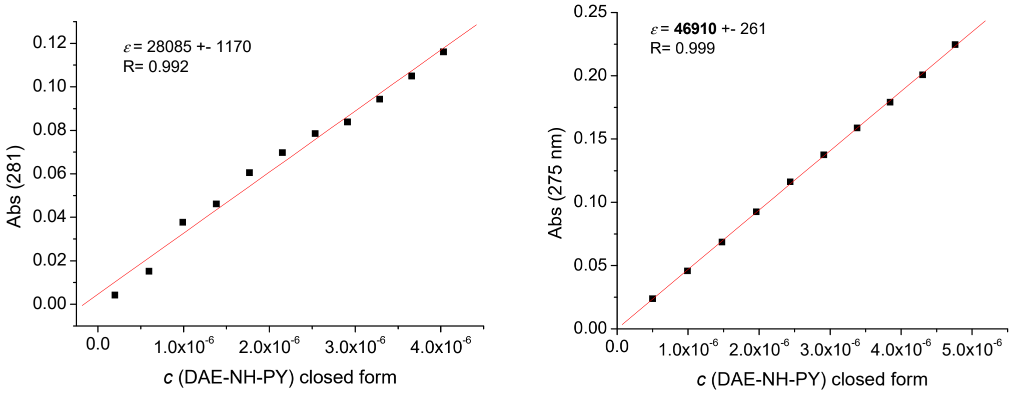


a) b)

**Figure S7.** Dependence of UV/Vis spectra on concentration increase of **DAE-NH-PY (closed form)** in **a)** sodium cacodylate buffer (pH = 7.0, *I* = 50 mM) / 4 % MeOH / 0.2 % DMSO **b)** in MeOH / 4 % DMSO

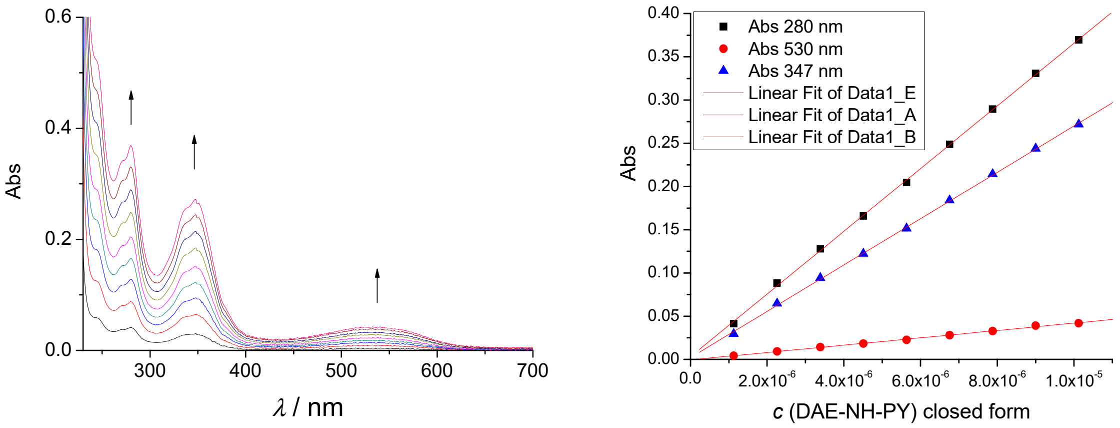


a) b)

**Figure S8. a)** Dependence of UV/Vis spectra on concentration of **DAE-NH-PY (closed form)** in sodium cacodylate buffer (pH = 7.0, *I* = 50 mM) / 0.5 % DMSO **b)** Dependence of UV/Vis spectra on concentration increase of **DAE-NH-PY (closed form)** in sodium cacodylate buffer (pH = 7.0, *I* = 50 mM) / 0.5 % DMSO

Photochemistry:

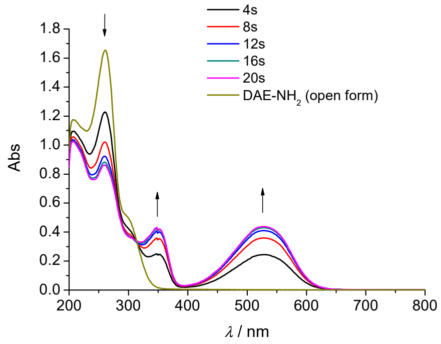


a)

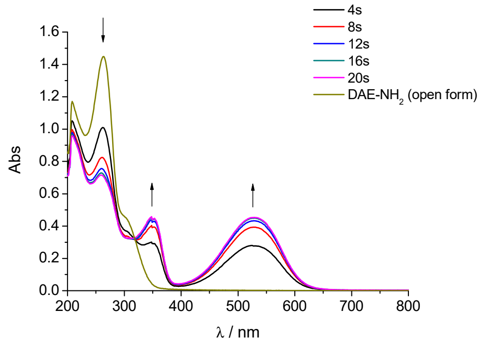


b)

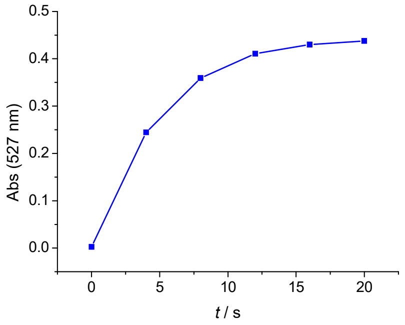


c)

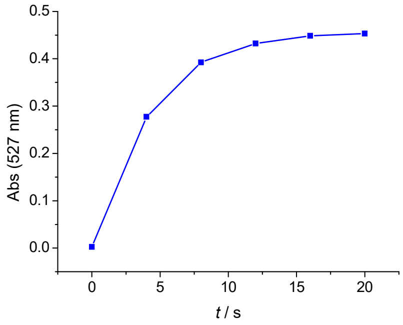


d)

**Figure S9.** Changes in UV/Vis spectra of **DAE-NH$_2$** ($c$ = 1.0x10$^{-4}$ M) during 64 W, range 254 -315 nm irradiation ($t_{tot.}$ = 20.00 s) **a)** in MeOH **b)** Na cacodylate buffer (pH = 7.0, *I* = 50 mM). Changes at λ = 527 nm as a function of irradiation time ($t_{tot.}$ = 20.00 s). Extrapolation demonstrated 100 % conversion in 20 sec: **c)** MeOH, **d)** Na cacodylate buffer (pH = 7.0, *I* = 50 mM)

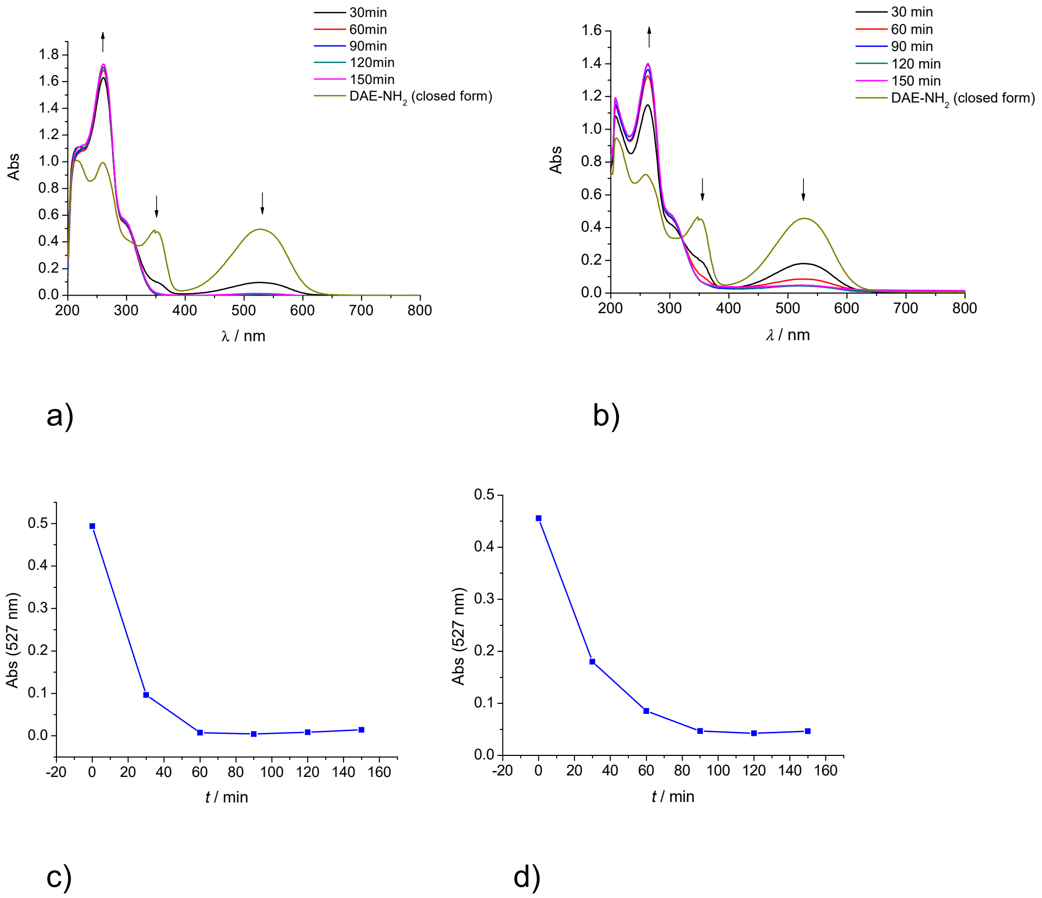


**Figure S10.** Changes in UV/Vis spectra of **DAE-NH₂** ($c$ = 1.0x $10^{-4}$) at different times of 64 W, range 400-700 nm lamps irradiation ($t_{tot}$. = 150.00 min), **a)** in MeOH, **b)** Na cacodylate buffer (pH = 7.0, $I$ = 50 mM). Changes at λ = 527 nm as a function of irradiation time ($t_{tot}$. = 150.00 min). Extrapolation demonstrated 100 % conversion in 150 min. **c)** in MeOH, **d)** Na cacodylate buffer (pH = 7.0, $I$ = 50 mM)

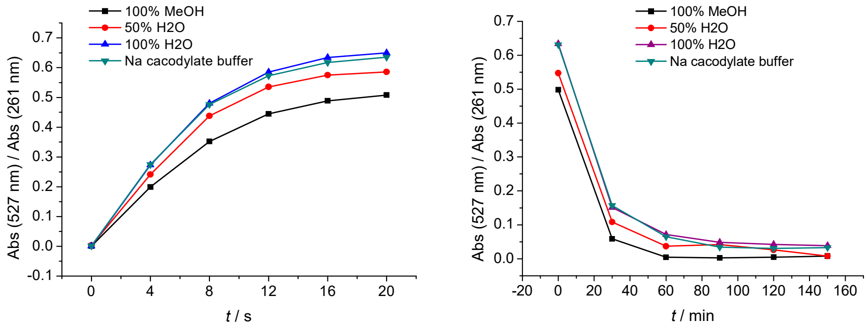


a) b)

**Figure S11.** Measurements of **DAE-NH₂** ($c$ = 1.0x10$^{-4}$ M) at MeOH, 50 % $H_2O$ in MeOH, $H_2O$ and Na cacodylate buffer (pH = 7.0*, I* = 50 mM) showing $A_{527}/A_{261}$ ratios durring **a)** irradiation with 64 W, range 254 - 315 nm lamps, as a function of irradiation time ($t_{tot.}$ = 20.00 s), **b)** irradiation with 64 W, range 400-700 nm lamps, as a function of irradiation time ($t_{tot.}$ = 150.00 min).

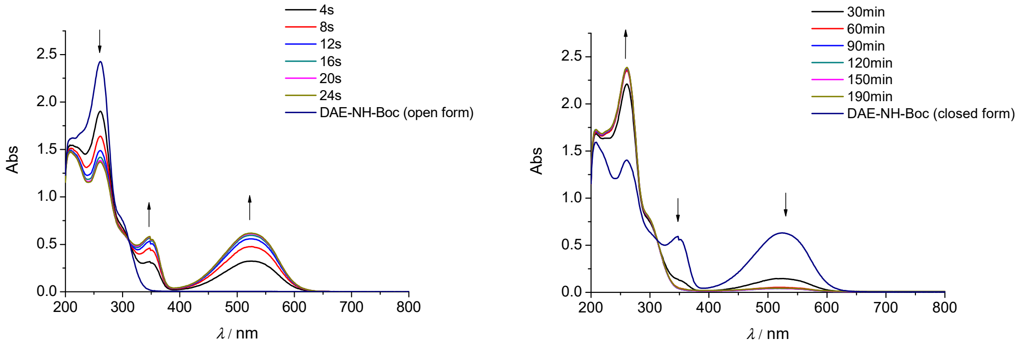


a) b)

**Figure S12.** Changes in UV/Vis spectra of **DAE-NH-Boc** ($c$ = 1.0x10$^{-4}$ M) in MeOH during **a)** 64 W, range 254-315 nm lamps irradiation ($t_{tot.}$ = 24.00 s), **b)** 64 W, range 400-700 nm lamps irradiation ($t_{tot}$ = 190.00 min)

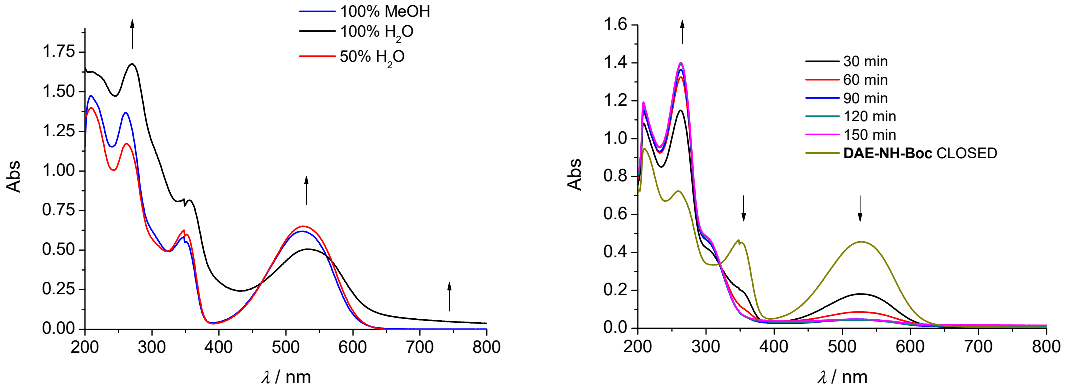


**Figure S13.** LEFT: Changes in UV/Vis spectra of **DAE-NH-Boc** ($c$ = 1.0x10$^{-4}$ M) after 64 W, range 254-315 nm lamps irradiation ($t_{tot}$. = 24.00 s) in MeOH, 100 % $H_2O$ and 50 % $H_2O$. RIGHT: De-cyclisation in Na cacodylate buffer (pH = 7.0, I = 50 mM), 64 W, range 400-700 nm lamps irradiation ($t_{tot}$ = 150.00 min)

Preparation of a working solution of **DAE-NH-PY** (open form) in Na cacodylate buffer or MeOH was done by already described procedure [3].

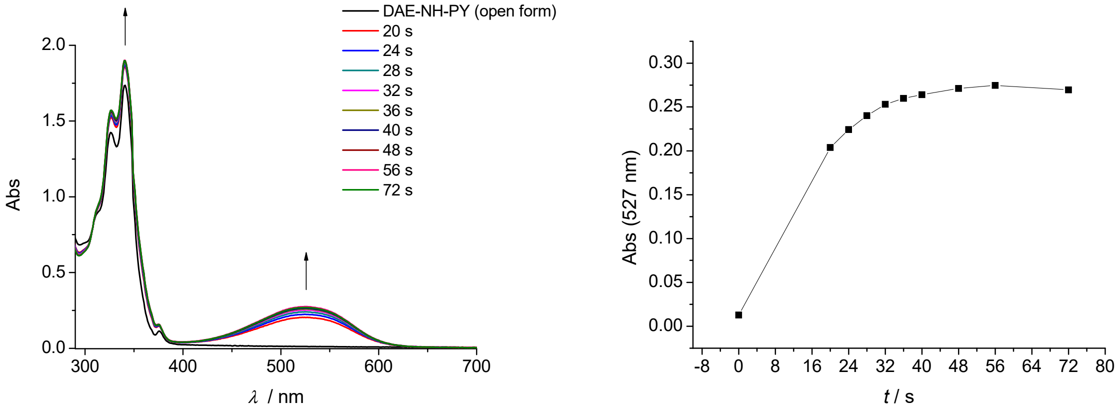


a) b)

**Figure S14. a)** Changes in UV/Vis spectra of **DAE-NH-PY** (***c* = 1.0x10$^{-4}$ M**) during 64 W, range 254-315 nm lamps irradiation ($t_{tot.}$ = 72.00 s) in MeOH / 4 % DMSO **b)** Changes at $\lambda$ = 527 nm of **DAE-NH-PY** (***c* = 1.0x10$^{-4}$ M**) in MeOH / 4 % DMSO, as a function of irradiation time. Extrapolation demonstrated 100 % conversion in 72 s.

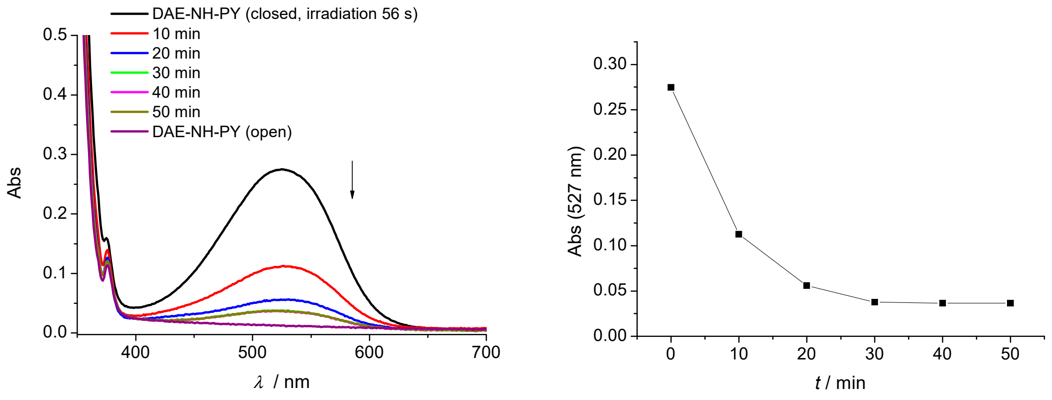


a) b)

**Figure S15. a)** Changes in UV/Vis spectra of **DAE-NH-PY** (***c* = 1.0x10$^{-4}$ M**) during 64 W, range 400 - 700 nm lamps irradiation ($t_{tot.}$ = 50.00 min) in MeOH / 4 % DMSO **b)** Changes at $\lambda$ = 527 nm of **DAE-NH-PY** (***c* = 1.0x10$^{-4}$ M**) in MeOH / 4 % DMSO, as a function of irradiation time. Extrapolation demonstrated 100 % conversion in 50 min.

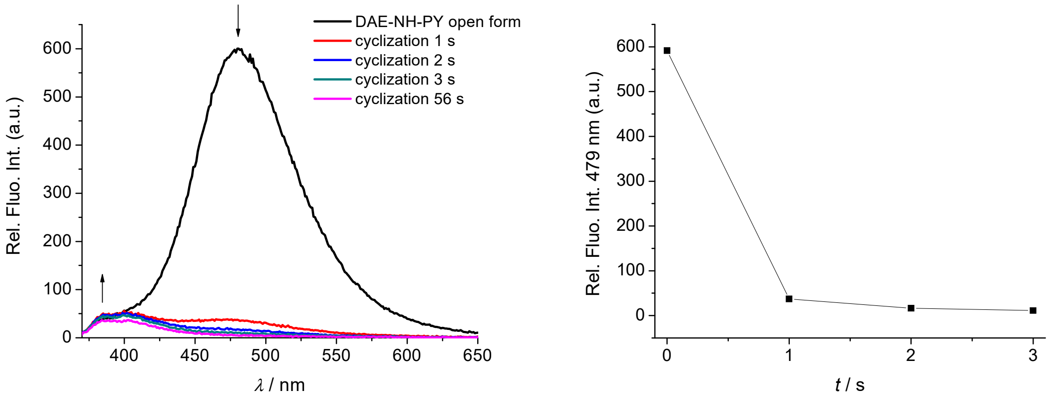


a) b)

**Figure S16. a)** Changes in emission spectra of **DAE-NH-PY** (***c* = 1.0x10$^{-6}$ M**) during 64 W, range 254-315 nm lamps irradiation ($t_{tot.}$ = 56.00 s) in Na cacodylate buffer (pH = 7.0, I = 50 mM) / MeOH (0.9 %) / DMSO (0.04 %), **b)** Changes in fluorescence emission intensity ($I_{fluo}$(479 nm) of **DAE-NH-PY** (***c* = 1.0x10$^{-6}$ M**) in Na cacodylate buffer (pH = 7.0, I = 50 mM) **/** MeOH (0.9 %) / DMSO (0.04 %), as a function of irradiation time.

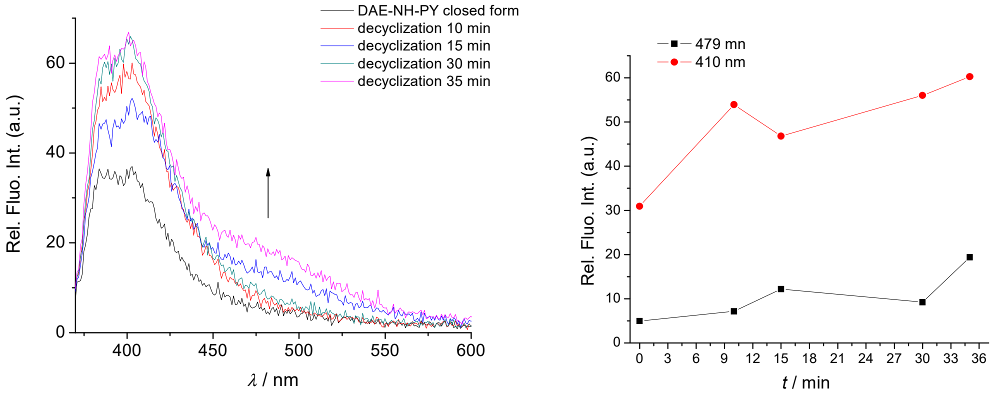


a) b)

**Figure S17. a)** Changes in emission spectra of cyclic form of **DAE-NH-PY** (*c* = 1.0x10$^{-6}$ M, as prepared in Figure 21.) during 64 W, range 400-700 nm lamps irradiation ($t_{tot.}$ = 35.00 min) in Na cacodylate buffer (pH = 7.0, I = 50 mM) / MeOH (0.9 %) / DMSO (0.04 %), **b)** Changes in fluorescence emission intensity (black: $I_{fluo}$ = 479 nm, red: $I_{fluo}$ = 479 nm) of **DAE-NH-PY** (***c* = 1.0x10$^{-6}$ M**) in Na cacodylate buffer (pH = 7.0, I = 50 mM) **/** MeOH (0.9 %) / DMSO (0.04 %), as a function of irradiation time.

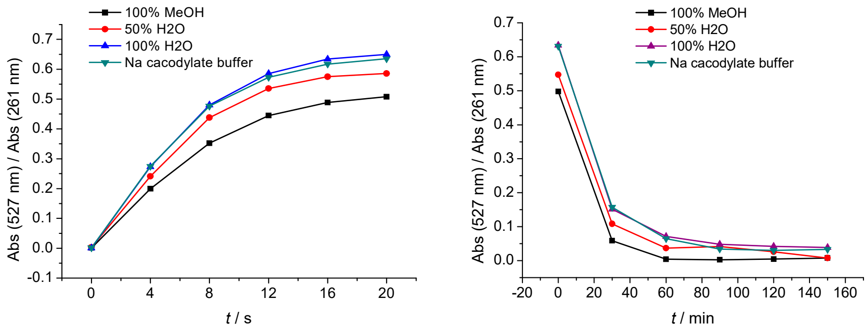


a) b)

**Figure S18.** Measurements of **DAE-NH-PY** ($c$ = 1.0x$10^{-4}$ M) at MeOH, 50 % $H_2O$ in MeOH, $H_2O$ and Na cacodylate buffer (pH = 7.0*, I* = 50 mM) showing $A_{527}/A_{261}$ ratios durring **a)** irradiation with 64 W, range 254 - 315 nm lamps, as a function of irradiation time ($t_{tot}$. = 20.00 s), **b)** irradiation with 64 W, range 400-700 nm lamps, as a function of irradiation time ($t_{tot}$. = 150.00 min).

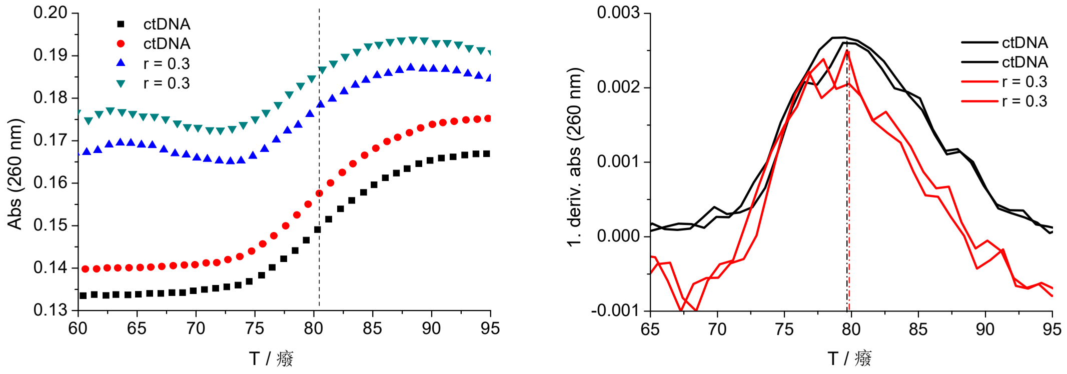


a) b)

**Figure S19. a)** Melting curve of ctDNA upon addition r = 0.3 ([compound/ [polynucleotide]) of **DAE-NH-PY (open form)** at pH 7.0 (buffer sodium cacodylate, *I* = 0.05 M) / 0.5 % DMSO, **b)** first derivation of absorbance on temperature.

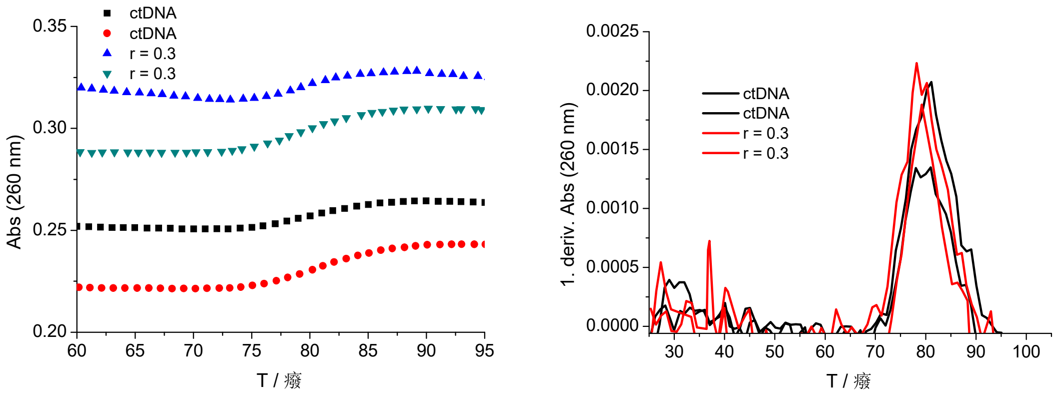


a) b)

**Figure S20. a)** Melting curve of ctDNA upon addition r = 0.3 ([compound/ [polynucleotide]) of **DAE-NH-PY (closed form)** at pH 7.0 (buffer sodium cacodylate, *I* = 0.05 M) / 0.2 % DMSO, **b)** first derivation of absorbance on temperature.

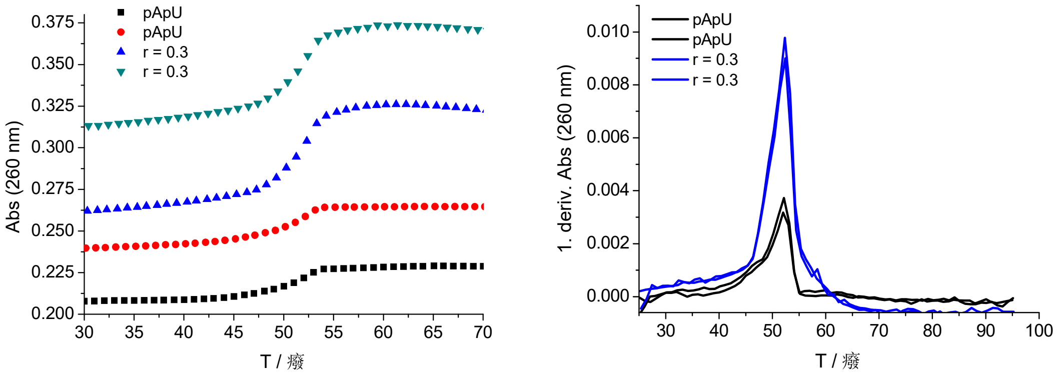


a) b)

**Figure S21. a)** Melting curve of pApU upon addition r = 0.3 ([compound/ [polynucleotide]) of **DAE-NH-PY (closed form)** at pH 7.0 (buffer sodium cacodylate, *I* = 0.05 M) / 4 % DMSO, **b)** first derivation of absorbance on temperature.

**Circular dichroism:**

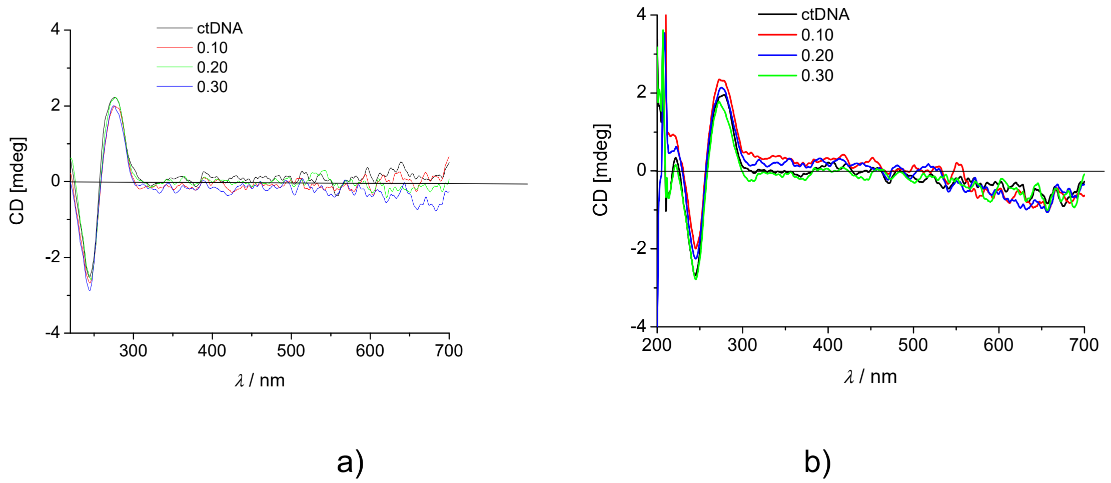


**Figure S22.** CD titration of ctDNA ($c$ = 2×10$^{-5}$ M) with a) OPEN **DAE-NH$_2$**, b) CLOSED **DAE-NH$_2$ ;** at molar ratios r = [compound] / [polynucleotide] in Na cacodylate buffer (pH = 7.0, I = 50 mM)

## Fluorimetric titrations:

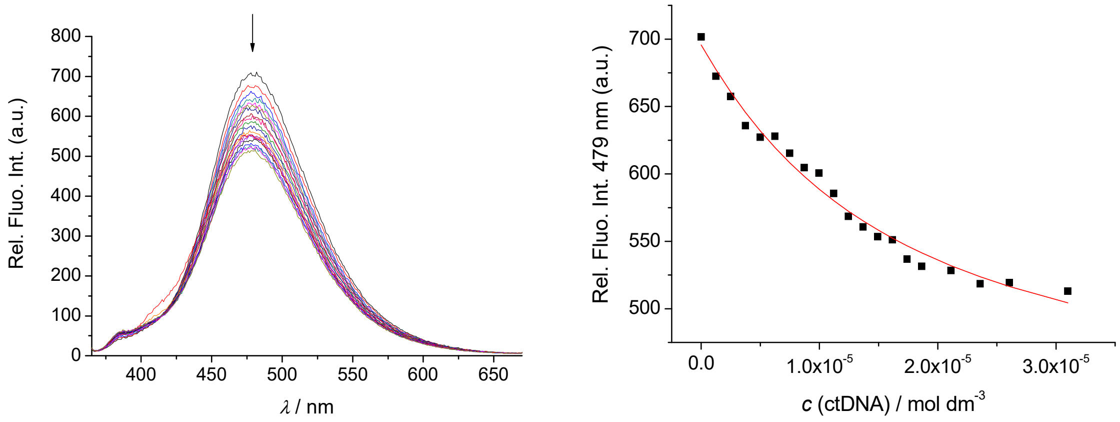


a) b)

**Figure S23. a)** Changes in fluorescence spectrum of **DAE-NH-PY** (open form, $c = 1.00 \times 10^{-6}$ M) upon titration with ctDNA; **b)** Dependence of **DAE-NH-PY** (open form) intensity at $\lambda_{max}$ = 479 nm on $c$(ctDNA), in Na cacodylate buffer (pH = 7.0, I = 50 mM) / 0.04 % DMSO (slit: 10-10, excitation: 348 nm, emission: 479 nm)

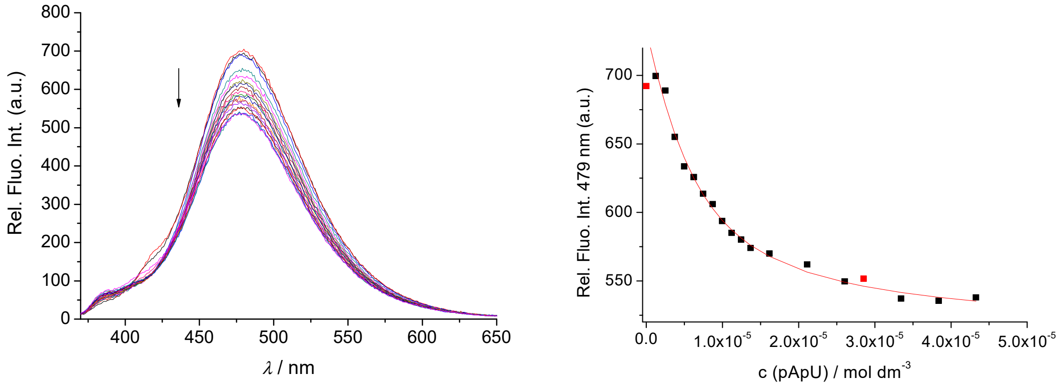


a) b)

**Figure S24. a)** Changes in fluorescence spectrum of **DAE-NH-PY** (open form, $c = 1.00\times 10^{-6}$ M) upon titration with pApU; **b)** Dependence of **DAE-NH-PY** (open form) intensity at $\lambda_{max}$ = 479 nm on $c$(pApU), in Na cacodylate buffer (pH = 7.0, I = 50 mM) / 0.04 % DMSO (slit: 10-10, excitation: 348 nm, emission: 479 nm)

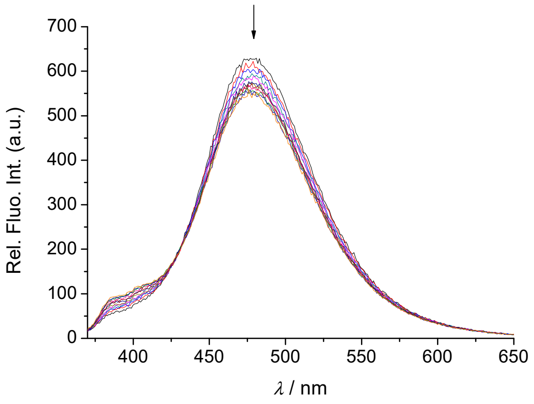


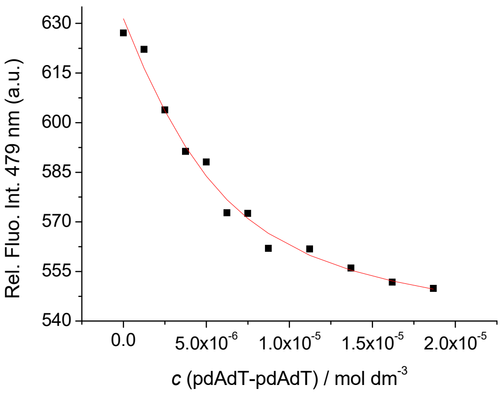


a) b)

**Figure S25. a)** Changes in fluorescence spectrum of **DAE-NH-PY** (open form, $c$ = $1.00\times10^{-6}$ M) upon titration with $p(dAdT)_2$; **b)** Dependence of **DAE-NH-PY** (open form) intensity at $\lambda_{max}$ = 479 nm on $c(p(dAdT)_2)$, in Na cacodylate buffer (pH = 7.0, I = 50 mM) / 0.04 % DMSO (slit: 10-10, excitation: 348 nm, emission: 479 nm)

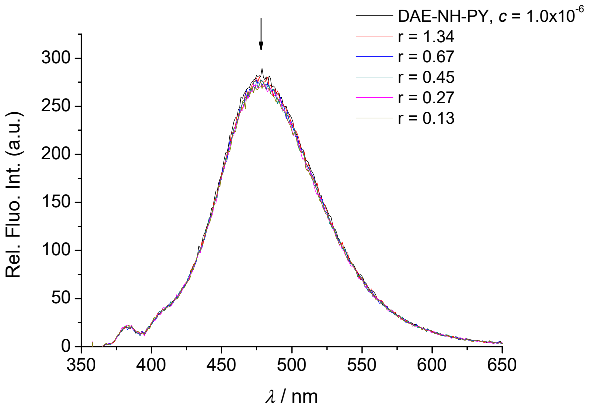


**Figure S26.** Changes in fluorescence spectrum of **DAE-NH-PY** (open form, $c$ = $1.00\times10^{-6}$ M) upon titration with $p(dGdC)_2$

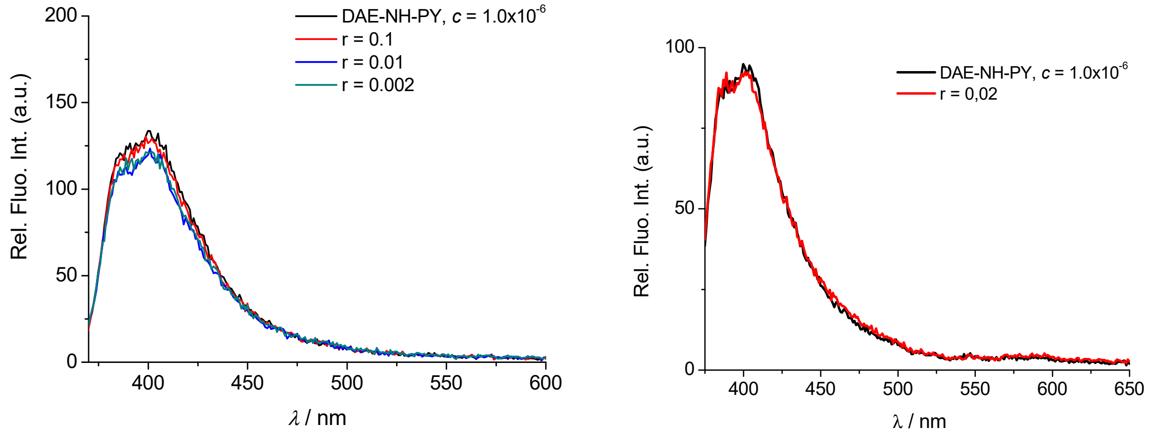


a) b)

**Figure S27.** Changes in fluorescence spectrum of **DAE-NH-PY** (closed form, $c$ = $1.00\times10^{-6}$ M) upon titration with a) ctDNA, b) pApU in Na cacodylate buffer (pH = 7.0*,* $I$ = 50 mM) / 0.04 % DMSO (slit: 10-10, excitation: 348 nm, emission: 400)

[1] D. J. van Dijken, J. M. Beierle, M. C. A. Stuart, W. Szymanski,W. R. Browne, B. L. Feringa, *Angew. Chem. Int. Ed*., 2014, **53**, 1.
[2] D. R. Arnold, C. P. Hadjiantoniou, *Can. J. Chem.,* 1978, **56**, 1970.
[3] G. M. Badger, H. J. Rodda, W. H. F. Sasse *J. Chem. Soc.,* 1954, 4162
[4] S. Tyrlik, I. Wolochowicz, *Bull. Soc. Chim., Fr.,* 1973, 2147
[5] S. Yamamura, M. Toda, Y. Hirata, *Organic Syntheses,* 1988, **6**, 289
[6] V. Dourtoglou, B. Gross, *Synthesis*, 1984, 572
[7] Z. J. Kaminski, *Tetrahedron Lett*., 1985, **26**, 2901.
[8] Chaires, J. B.; Dattagupta, N.; Crothers, D. M. *Biochemistry,* 1982, **21**, 3933-3940.
[9] Saenger, W. *Principles of Nucleic Acid Structure*; Springer-Verlag, New York, 1983.
[10] Cantor, C. R.; Schimmel, P. R. *Biophysical Chemistry* 1980, **3**, 1109-1181.
[11] S. Hartwig, M. M. Nguyen, S. Hecht, *Polym. Chem*., 2010, **1**, 69.
